# Targeting Ikaros and Aiolos with pomalidomide fails to reactivate or induce apoptosis of the latent HIV reservoir

**DOI:** 10.1128/jvi.01676-24

**Published:** 2025-02-04

**Authors:** Rachel D. Pascoe, Youry Kim, Ajantha Rhodes, Jesslyn Ong, Carolin Tumpach, Celine Gubser, J. Judy Chang, James H. McMahon, Sharon R. Lewin, Thomas A. Rasmussen

**Affiliations:** 1 Department of Microbiology and Immunology, The University of Melbourne at the Peter Doherty Institute for Infection and Immunity 2281 , Melbourne, Victoria, Australia; 2 Department of Infectious Diseases, Aarhus University Hospital 11297 , Aarhus, Denmark; 3 Department of Infectious Diseases, The University of Melbourne at the Peter Doherty Institute for Infection and Immunity 2281 , Melbourne, Victoria, Australia; 4 Department of Infectious Diseases, Alfred Hospital and Monash University 589641 , Melbourne, Victoria, Australia; 5 Victorian Infectious Diseases Service, Royal Melbourne Hospital at the Peter Doherty Institute for Infection and Immunity 90134 , Melbourne, Victoria, Australia; Icahn School of Medicine at Mount Sinai, New York, New York, USA

**Keywords:** HIV, latency, apoptosis

## Abstract

HIV persists in people living with HIV (PLHIV) on antiretroviral therapy (ART) in long-lived and proliferating latently infected CD4+ T cells that selectively express pro-survival proteins, including the zinc finger proteins, Ikaros and Aiolos. In this study, we investigated whether pomalidomide, an immunomodulatory agent that induces degradation of Ikaros and Aiolos, could increase the death of HIV-infected cells and/or reverse HIV latency. Using an *in vitro* model of CD4+ T cells infected with a green fluorescent protein (GFP) reporter virus, pomalidomide increased the expression of the pro-survival protein B cell lymphoma (Bcl)-2 and did not increase apoptosis of GFP+ HIV productively infected CD4+ T cells. Pomalidomide also increased the expression of CD155 and UL16-binding protein (ULBP) stress proteins on GFP+ HIV productively infected CD4+ T cells, but this did not translate to enhanced clearance following co-culture with a natural killer (NK) cell line. Using CD4+ T cells from PLHIV on ART, pomalidomide *ex vivo* activated memory CD4+ T cells resulting in elevated HLA-DR expression and induced CD4+ T cell proliferation but only in the presence of T cell receptor stimulation with anti-CD3 and anti-CD28. There was no effect on cell-associated HIV RNA or the frequency of intact HIV DNA. In conclusion, despite an increase in stress protein expression, promoting Ikaros and Aiolos degradation in CD4+ T cells using pomalidomide did not directly induce apoptosis of HIV-infected cells or induce HIV latency reversal.

**IMPORTANCE:**

People living with HIV (PLHIV) require lifelong antiretroviral therapy (ART) due to the persistence of latently infected cells. The zinc finger proteins, Ikaros and Aiolos, have recently been implicated in promoting the persistence of latently infected cells. In this study, we investigated the effects of pomalidomide, an immunomodulatory imide drug that induces the degradation of Ikaros and Aiolos, on HIV latency reversal and death of infected cells. Using CD4+ T cells from people living with HIV on suppressive antiretroviral therapy, as well as an *in vitro* model of productive HIV infection, we found that pomalidomide induced T cell activation and expression of stress proteins but no evidence of latency reversal or selective death of infected cells.

## INTRODUCTION

Despite the great success of antiretroviral therapy (ART) in reducing morbidity and mortality in people living with HIV (PLHIV), treatment is needed lifelong due to the persistence of latent HIV in long-lived and proliferating CD4+ T cells. Latency occurs when the virus integrates into the host genome but there is no or minimal virus transcription and, therefore, no protein expression (1). Latently infected cells can evade immune-mediated clearance due to minimal or lack of viral antigen presentation (2, 3), the expression of immune checkpoint (IC) proteins that can reduce HIV transcription and help evade immunity (4–6), and through elevated expression of pro-survival proteins (7–9). The transcription factors, Aiolos (IKZF3) and Ikaros (IKZF1), have also been recently implicated in the survival of transcriptionally active HIV-infected CD4+ T cells (10) and maintenance of latency via repression of HIV transcription (11), respectively. Here, we investigated the impact of pomalidomide, a third-generation immunomodulatory imide drug that has both immune-stimulatory and antiproliferative properties (12–16), and leads to the ubiquitination and degradation of Aiolos and Ikaros (14), on the HIV reservoir.

HIV latently infected cells persist in PLHIV on ART through several mechanisms, including the upregulation of pro-survival proteins and active repression of transcription (8, 10). The pro-survival protein, B cell lymphoma-2 (Bcl-2), is expressed on latently infected cells that survive exposure to HIV-specific cytotoxic CD8+ T cell clones (8), and treatment with the Bcl-2 antagonist, venetoclax, can deplete the HIV reservoir and delay time to viral rebound in an HIV humanized mouse model (9). Additionally, the expression of Baculoviral IAP Repeat Containing 5 (BIRC5), an inhibitor of apoptosis protein (IAP), was shown to be increased in clonally expanded cells harboring intact HIV, thereby blocking caspase-9-mediated apoptosis (7, 8). The transcription factor, Ikaros, was recently shown to support HIV latency through transcriptional repression of the HIV-1 long-terminal repeat (LTR) by inducing a repressive chromatin state (11) and suppressing HIV reactivation following stimulation (17, 18). The transcription factor, Aiolos, has also been implicated in the survival of transcriptionally active HIV-infected CD4+ T cells and was shown to be elevated in both latent and transcriptionally active HIV-infected cells from PLHIV on ART (10). Disrupting these pro-survival mechanisms may support a reduction of the HIV reservoir as part of a curative strategy for PLHIV on suppressive ART.

Pomalidomide and lenalidomide are derivatives of thalidomide and engage with cereblon (CBRN), a highly conserved E3 ubiquitin ligase (14, 19). This interaction leads to the ubiquitination and degradation of transcriptional regulators, Ikaros and Aiolos (14). Loss of Ikaros and Aiolos induces a potent IL-2 response, with subsequent stimulation of T cells and natural killer (NK) cells through the phosphatidylinositol 3-kinase (PI3K)/AKT/mechanistic target of rapamycin (mTOR) pathways (20–22). Pomalidomide has well-described antiproliferative and pro-apoptotic properties, which have been defined in the setting of malignancy and chronic viral infection. Pomalidomide can upregulate tumor suppressor genes, namely p21^WAF-1^, to induce cell cycle arrest (23–25), and induce caspase-8 and sensitize cells to Fas-induced apoptosis (26, 27). In addition, pomalidomide can sensitize virus-infected cells to immune-mediated clearance through upregulating the expression of the stress protein, UL16-binding protein (ULBP)-1, and/or upregulating major histocompatibility complex (MHC)-1 expression in cell line models for multiple myeloma, Burkitts lymphoma, Epstein-Barr Virus (EBV), and in human T cell leukemia virus-type 1 (HTLV-1)-infected primary T cells (28, 29). Pomalidomide and its analog, iberdomide, have also been shown to reactivate HIV from latency, with both agents found to induce HIV RNA transcription *ex vivo* in CD4+ T cells isolated from ART-suppressed PLHIV but only at a much higher concentration than what can be achieved therapeutically (11).

We hypothesized that pomalidomide will enhance the susceptibility of infected cells to undergo apoptosis through the degradation of Aiolos and Ikaros. We, therefore, investigated the effects of pomalidomide at clinically relevant concentrations *ex vivo* on the HIV reservoir. We found pomalidomide did not selectively induce apoptosis in *in vitro* HIV productively infected CD4+ T cells, with an upregulation of Bcl-2 following pomalidomide treatment, potentially protecting against apoptosis. Pomalidomide increased ULBP expression on HIV productively infected CD4+ T cells, but this did not translate to greater sensitivity to NK cell clearance using an NK cell line. Furthermore, while pomalidomide treatment induced activation of memory CD4+ T cells, pomalidomide did not induce latency reversal nor alter the frequency of cells harboring intact HIV provirus. Thus, pomalidomide did not demonstrate pro-apoptotic or latency reversal properties.

## RESULTS

### Pomalidomide upregulates Bcl-2 in HIV productively infected CD4+ T cells

In this study, pomalidomide was predominantly assessed *ex vivo* at 0.25 µM to reflect the maximal plasma concentration (C_MAX_) in HIV-negative individuals and PLHIV who received 5 mg pomalidomide daily for Kaposi Sarcoma, which at this dose was well tolerated with minimal serious adverse effects (30). Using antibodies to Ikaros (IKZF1) and Aiolos (IKZF3), we confirmed that treatment with pomalidomide *ex vivo* at 0.25, 2, and 10 µM induced potent degradation of Ikaros and Aiolos in CD4+ T cells from ART-suppressed PLHIV following 24 hours of treatment, and this degradation profile was maintained following 72 hours of continuous culture with pomalidomide *ex vivo* (Fig. S1).

**Fig 1:**
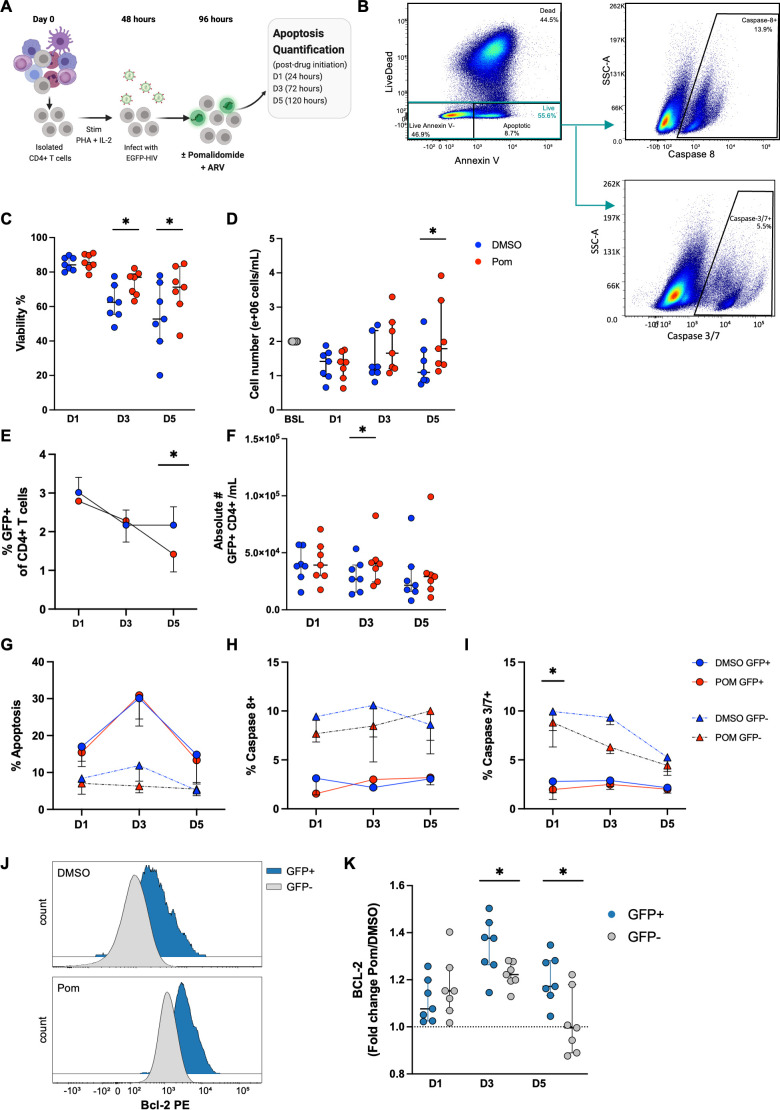
Pomalidomide upregulates Bcl-2, protecting HIV-infected cells from apoptosis. (**A**) Schematic of the HIV productive infection assay, where isolated CD4+ T cells were stimulated with phytohemagglutinin (PHA) and IL-2 before *in vitro* infection with green fluorescent protein (GFP)-expressing HIV, and treated with pomalidomide at 0.25 µM, or DMSO, for 5 days. (**B**) Representative flow plot for viability gating and caspase gating after apoptosis quantification staining. Data were analyzed after 1, 3, and 5 days of culture with DMSO (blue) or pomalidomide (red) and then analyzed for (**C**) frequency of viable (live) CD4+ T cells; (**D**) absolute number of CD4+ T cells; (**E**) frequency of HIV productively infected (GFP+) CD4+ T cells; (**F**) absolute number of HIV productively infected (GFP+) CD4+ T cells; (**G**) frequency of apoptotic (Live/Dead-Annexin V+) cells within the HIV productively infected (GFP+) and HIV-uninfected/non-productively infected (GFP–) CD4+ T cell populations; (**H**) frequency of caspase-8+ cells within the live GFP+ and GFP– CD4+ T cell populations; (**I**) frequency of caspase-3/7+ cells within the live GFP+ and GFP– CD4+ T cell populations. (**J**) Representative histogram overlay of B cell lymphoma (Bcl)-2 expression in HIV productively infected (GFP+) and HIV-uninfected/non-productively infected (GFP–) CD4+ T cell populations following DMSO or pomalidomide treatment for 3 days; (**K**) Bcl-2 mean fluorescence index (MFI) within the live GFP+ and GFP– CD4+ T cell populations following pomalidomide treatment for 5 days, relative to DMSO treatment. Each symbol in panels C, D, F, and K represents a single donor. Each symbol in panels E and G–I represents the average of seven donors. *P < 0.05; ns, not significant. Wilcoxon matched-pairs signed-rank test. Bars show median + IQR. Schematic created with BioRender.com.

To investigate if pomalidomide enhanced apoptosis of productively infected CD4+ T cells, an *in vitro* HIV productive infection assay was used. CD4+ T cells from an HIV-negative donor were infected with an R5-tropic replication-competent enhanced green fluorescent protein (GFP)-reporter HIV and treated with pomalidomide at 0.25 µM (Fig. 1A). Compared to dimethyl sulfoxide (DMSO), pomalidomide increased the viability of total CD4+ T cells by 1.2-fold and 1.4-fold after 3 and 5 days of treatment, respectively, with a corresponding 1.6-median-fold increase in the absolute number of CD4+ T cells on day 5 (Fig. 1B through D). Pomalidomide reduced the frequency of GFP+ CD4+ T cells after 5 days of treatment (Fig. 1E), consistent with a reduction in HIV productively infected CD4+ T cells. There was no change in the absolute number of GFP+ CD4+ T cells following pomalidomide treatment (Fig. 1F), suggesting that the reduction in the percentage of GFP+ CD4+ T cells was a result of the expansion of GFP– CD4+ T cells following pomalidomide treatment, which predominately comprises HIV-uninfected CD4+ T cells, but may also contain non-productively infected CD4+ T cells and abortively infected CD4+ T cells (31). These data suggest that pomalidomide may have a different effect on the survival and/or proliferation of uninfected and productively infected CD4+ T cells.

Cells undergoing apoptosis were identified by the expression of Annexin V, a measure of phosphatidylserine (PtdSer) expression on the outer plasma membrane leaflet, and negative expression for live/dead stain (Fig. 1B). Compared to GFP– cells, GFP+ HIV productively infected CD4+ T cells expressed higher levels of apoptosis (defined as Live/Dead stain-Annexin V+) with a 2.3-median-fold increase on day 3 in the DMSO control (Fig. 1G) with no further increase in the presence of pomalidomide. As pomalidomide has previously been shown to induce apoptosis through caspase-8 (26, 27), and PtdSer expression is mediated by active-caspase-3/7 inhibition of PtdSer flippase (32), we next quantified the expression of activated caspase-3, -7, and -8. We found that pomalidomide did not significantly alter the expression of activated caspase-8 in either GFP+ or GFP– CD4+ T cells at any timepoint evaluated, nor was pomalidomide treatment associated with any changes in caspase-3/7 expression in GFP+ cells (Fig. 1H and I). Notably, the expression of activated caspase-3, -7, and -8 was greater in GFP– CD4+ T cells relative to GFP+ CD4+ T cells, despite apoptosis (determined by Annexin V staining) being greater in GFP+ CD4+ T cells at all timepoints measured. These findings may reflect caspase-independent apoptosis or pro-survival mechanisms that support the persistence of the HIV productively infected CD4+ T cells.

To further explore pro-survival factors that may influence the apoptosis-promoting effects of either pomalidomide or HIV infection, we evaluated the expression of the pro-survival protein, Bcl-2, which has previously been implicated in HIV persistence (8). Aiolos, the transcription factor degraded by pomalidomide, has been shown to bind and promote the expression of Bcl-2 proteins (33), and the HIV transactivating response element (TAR) miRNA has been implicated in the upregulation of Aiolos (34); hence, pomalidomide was investigated as an agent to disrupt Bcl-2-mediated persistence. Bcl-2 expression was higher in GFP+ HIV productively infected CD4+ T cells compared to GFP– CD4+ T cells (Fig. 1J). Compared to DMSO, pomalidomide significantly upregulated Bcl-2 by 1.38-fold and 1.17-fold in GFP+ CD4+ T cells *ex vivo* after 3 and 5 days of treatment, respectively, and in GFP– cells by 1.15- and 1.22-fold after 1 and 3 days of treatment, respectively, but this returned to baseline levels by day 5 (Fig. 1K). Pomalidomide-associated Bcl-2 upregulation was significantly higher in GFP+ compared to GFP– cells, with 1.1- and 1.2-fold greater upregulation by day 3 and 5, respectively. Collectively, these data demonstrated that in productive infection, pomalidomide increased the expression of Bcl-2 and did not induce apoptosis, contrary to the anticipated effects of the drug.

### Pomalidomide upregulates CD155 and ULBP on productive HIV-infected CD4+ T cells

**Fig 2:**
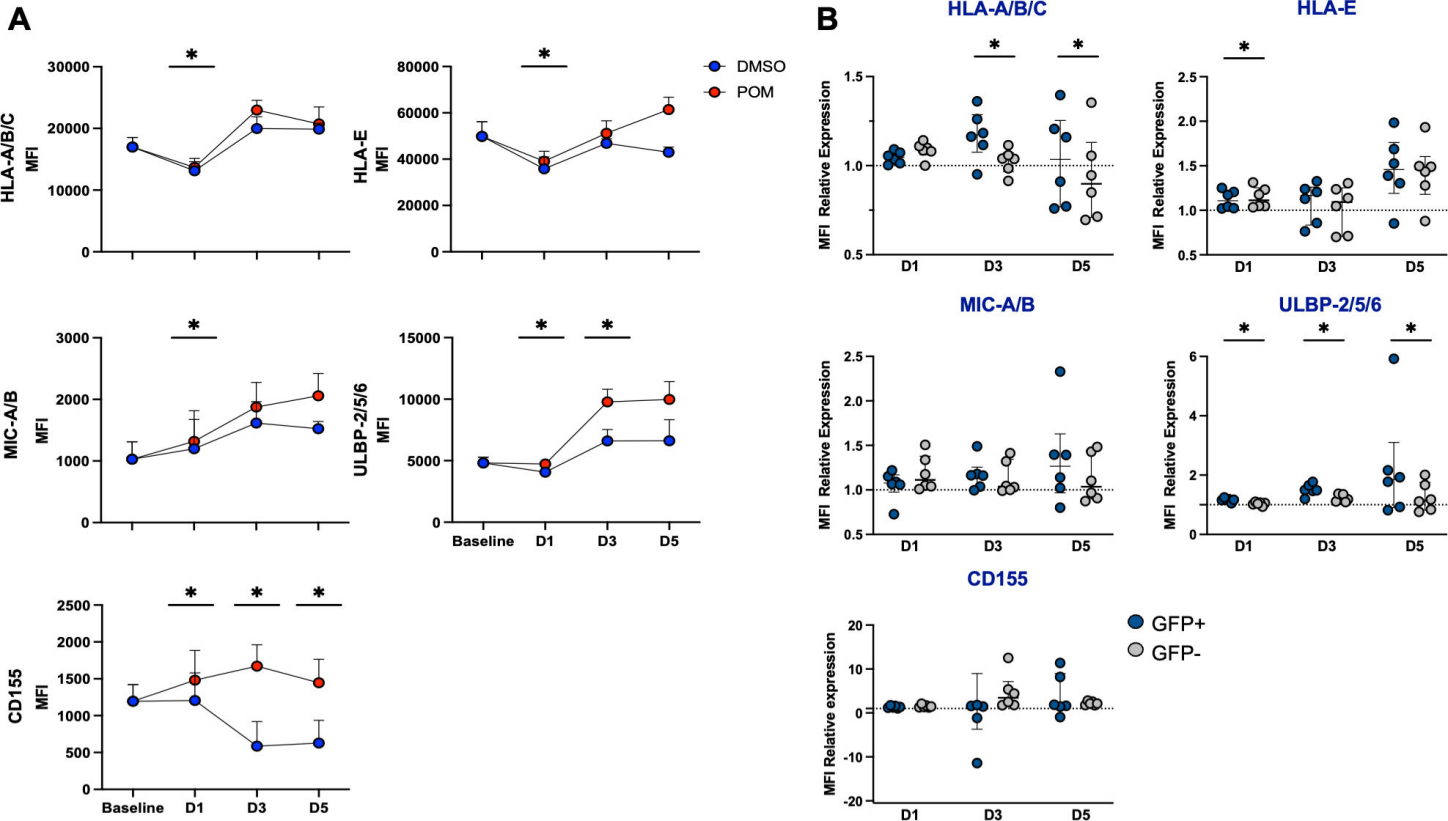
Pomalidomide upregulates CD155 and ULBP in HIV infected. (**A**) Following infection *in vitro* of CD4+ T cells with a green fluorescent protein (GFP)-expressing virus, cells were treated with pomalidomide at 0.25 µM, or DMSO, for 5 days. Data were analyzed after 1, 3, and 5 days of culture with DMSO (blue) or pomalidomide (red) measuring the median fluorescence index (MFI) of HLA-A/B/C, HLA-E, MIC-A/B, ULBP-2/5/6, and CD155 expression on *in vitro*-infected CD4+ T cells, and then analyzed for (**B**) MFI relative expression on pomalidomide-treated HIV productively infected (GFP+) and HIV-uninfected/non-productively infected (GFP–) CD4+ T cells, relative to DMSO, with statistics performed between the GFP+ and GFP– populations. Each symbol in panel A indicates the average of six donors, and each symbol in panel B represents a single donor. *P < 0.05; ns, not significant. Wilcoxon matched-pairs signed-rank test. Bars show median + IQR.

To elucidate the susceptibility of HIV-infected cells to immune-mediated killing, *in vitro* infected CD4+ T cells were treated with pomalidomide or DMSO in the same HIV productive infection assay described (Fig. 1A). Consistent with what has been shown previously (35), productive HIV infection was associated with a downregulation of HLA-A/B/C and an increase in the median fluorescence of the stress proteins ULBP-2/5/6 (Fig. S2A and B) that, like MHC-I chain-related proteins (MIC)-A/B, engage the activating NK cell ligand NKG2D to induce NK cell cytotoxicity (36–38). Other markers evaluated included HLA-E and CD155, which have both been shown to be upregulated in HIV-infected cells (6). HLA-E engages with the NK cell inhibitory marker, NKG2A, and the NK cell activating marker, NKG2C, to facilitate the triggering of NK cell responses (39, 40). CD155 is a marker that engages either the inhibitory marker, T cell immunoreceptor with immunoglobulin and ITIM domains (TIGIT), to impair NK cells and CD8+ T cell responses, or DNAM-1 to induce NK cell responsiveness (41–43). Treating infected cells with pomalidomide led to a minor but significantly increased expression of HLA-A/B/C, HLA-E, MIC-A/B, ULBP-2/5/6, and CD155 on GFP+ HIV productively infected CD4+ T cells after 24 hours of treatment (Fig. 2A). Compared to DMSO, pomalidomide increased the expression of ULBP-2/5/6 by 1.5-fold on day 3, and CD155 was upregulated by 3.8-fold and 3.9-fold on days 3 and 5, respectively (Fig. 2A).

To evaluate the specificity of the effects on HIV productively infected CD4+ T cells, a direct comparison was performed between the pomalidomide-induced effects on GFP+ HIV productively infected CD4+ T cells relative to that on GFP– CD4+ T cells. Pomalidomide upregulated HLA-A/B/C, HLA-E, MIC-A/B, and CD155 on GFP– CD4+ T cells after 24 hours of treatment, and upregulated ULBP-2,5,6 after 3 days of treatment (Fig. S2C). Pomalidomide enhancement of ULBP-2/5/6 expression was significantly greater on productively HIV-infected CD4+ T cells than that on the GFP– fraction, which are predominately HIV-uninfected CD4+ T cells, with 1.24-fold and 1.62-fold greater upregulation of ULBP-2/5/6 on GFP+ relative to GFP– following pomalidomide treatment for 3 and 5 days, respectively (Fig. 2B). By contrast, there were no differences in the effects of pomalidomide on CD155 expression between GFP+ and GFP– cells.

To evaluate whether the increased expression of ULBP might translate to enhanced sensitivity to NKG2D-mediated NK cell lysis of HIV-infected cells, pomalidomide-treated CD4+ T cells were co-cultured with the NK cell line, KHYG-1, at an effector:target (KHYG-1:CD4+ T cell) ratio of 1:1 (Fig. S2D). Pomalidomide did not alter the sensitivity of HIV productively infected CD4+ T cells to NK cell killing, as indicated by a minimal reduction in the frequency of GFP+ cells (Fig. S2E). The addition of an anti-NKG2D blocking antibody did not change the clearance of GFP+ cells, reinforcing our findings that the upregulation of ULBP-2/5/6 and subsequent NKG2D interactions following pomalidomide treatment did not lead to a reduction of HIV productively infected cells.

Collectively, these data demonstrated that although pomalidomide upregulated CD155 and ULBP-2/5/6 in CD4+ T cells, with greater upregulation of ULBP-2/5/6 on GFP+ compared to GFP– CD4+ T cells, this did not result in enhanced killing of HIV productively infected CD4+ T cells through NKG2D interactions.

### Pomalidomide alters the expression of exhaustion and senescence markers on CD4+ T cells from PLHIV on ART

To determine the effect of pomalidomide on the HIV reservoir, we next examined the expression of IC markers known to be associated with HIV persistence (4–6), with programmed cell death protein-1 (PD-1), PD-1 ligand (PD-L1), and TIGIT reported to be elevated in clonally expanded latently infected cells containing intact HIV DNA (5, 6, 44). The immune senescence marker, CD57, and CD155 were also evaluated on CD4+ T cells from PLHIV on ART treatment with pomalidomide or DMSO *ex vivo* for 72 hours. Leukapheresis was performed on PLHIV on suppressive ART for at least 3 years. The clinical characteristics of PLHIV study participants are shown in Table S1. The participants had a median CD4+ T cell count at enrolment of 675.5 cells/mL, a median nadir CD4+ T cell count of 230 cells/µL, and a median duration of suppressed plasma HIV RNA of 10.7 years. Additional patient demographics and ART regimens are listed in Table S1.

**Fig 3:**
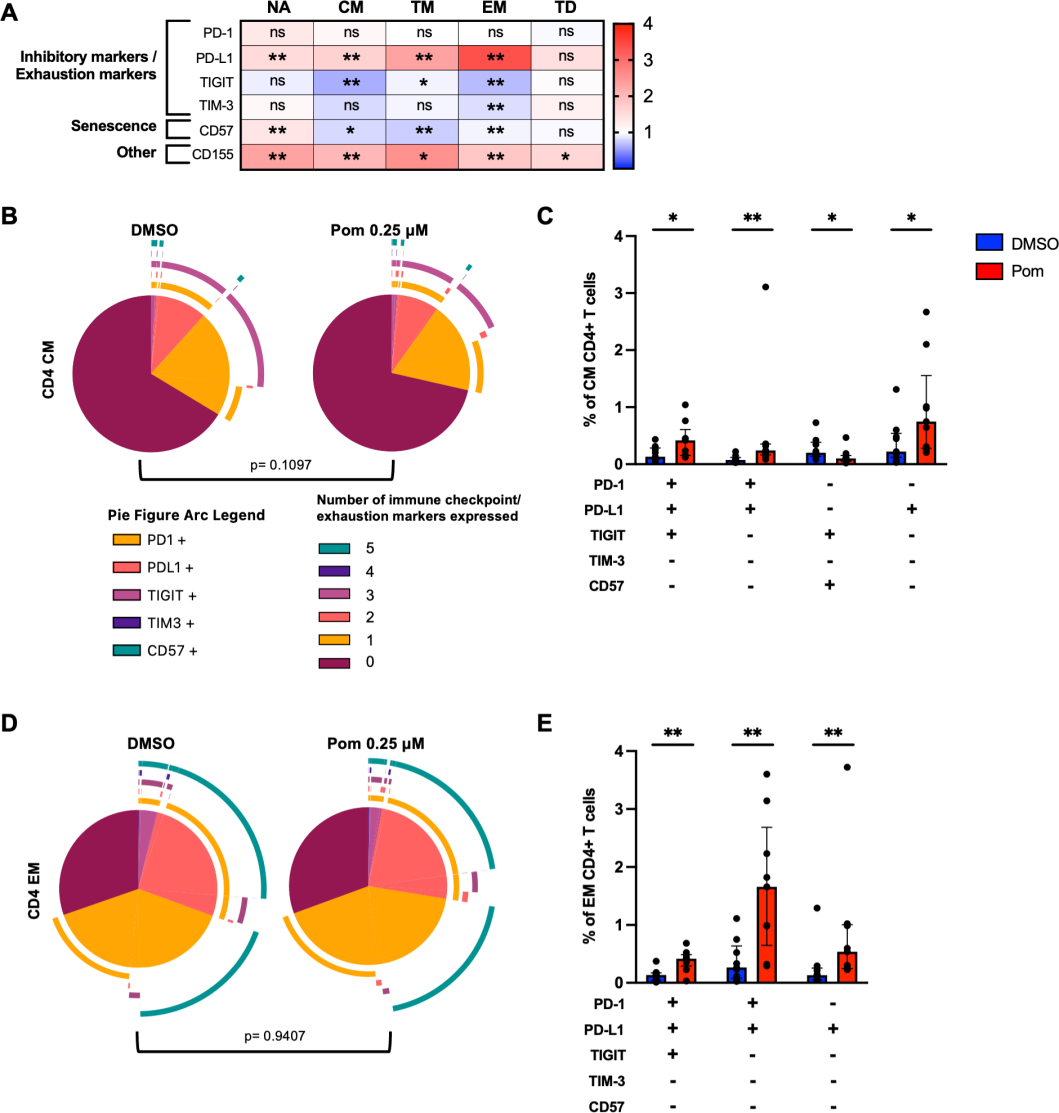
Pomalidomide alters the immune checkpoint, exhaustion, and immune senescence profile of effector memory CD4+ T cells from PLHIV treated *ex vivo*. PBMC from ART-suppressed PLHIV were treated with pomalidomide at 0.25 µM, or DMSO, for 72 hours *ex vivo*, and expression of immune checkpoint (IC) markers, immune senescence, and CD155 on CD4+ T cell subsets following pomalidomide treatment relative to DMSO control were measured using flow cytometry. (**A**) Comparisons in expression of markers in naïve (N), central memory (CM), transitional memory (TM), effector memory (EM), and terminally differentiated (TD) cells treated with pomalidomide compared to DMSO are shown as a heatmap. Red indicates increased expression, and blue indicates reduced expression with pomalidomide treatment relative to DMSO, with a scale of 1–4 representing the fold change. (**B**) Co-expression of different IC markers/exhaustion markers and immune senescence in DMSO-treated and pomalidomide-treated CM CD4+ T cells (internal slices), and the combinations of IC/exhaustion/senescence markers (external arcs). (**C**) Frequency of IC/exhaustion/senescence populations of CM CD4+ T cells. Of the 32 IC/exhaustion/senescence marker combinations, only statistically significant population changes are shown. (**D**) Average co-expression of different IC markers/exhaustion markers and immune senescence in DMSO-treated and pomalidomide-treated EM CD4+ T cells (internal slices), and the combinations of IC/exhaustion/senescence markers (external arcs). (**E**) Frequency of IC/exhaustion/senescence populations of EM CD4+ T cells. Of the 32 IC/exhaustion/senescence marker combinations, only statistically significant population changes are shown. For panels A, C, and E, statistical significance (*P* < 0.05) was determined using Wilcoxon matched-pairs signed-rank test. For panels B and D, statistical significance (*P* < 0.05) was determined by permutation tests. *P < 0.05; **P < 0.01; ns, not significant. Bars show median + IQR.

Compared to DMSO, pomalidomide significantly reduced the expression of TIGIT (median fold change [MFC] = 0.66) and T cell immunoglobulin and mucin domain-containing protein-3 (TIM-3) (MFC = 0.83), and reduced the expression of CD57 (MFC = 0.94) on effector memory (EM) CD4+ T cells (Fig. 3A; Fig. S3A through C), a population previously shown to be enriched with intact HIV in PLHIV on ART (45, 46). Pomalidomide did not induce any change in PD-1 expression but upregulated PD-L1 on naïve (NA), central memory (CM), transitional memory (TM), and EM CD4+ T cells (NA MFC = 1.53, CM MFC = 1.65, TM MFC = 2.32, EM MFC = 3.35) (Fig. 3A; Fig. S3A through C). There was no significant effect of pomalidomide on the overall number of IC or senescence markers expressed within CM or EM (Fig. 3B through E), but pomalidomide treatment did change the expression of a combination of markers, most pronounced for combinations that included PD-L1, consistent with the overall increased PD-L1 expression observed in all memory CD4+ T cell subsets. Collectively, pomalidomide reduced the expression of TIGIT and TIM-3 on CD4+ T cells, specifically in EM, while enhancing the expression of PD-L1. The enhanced expression of PD-L1 on HIV-infected target cells could enhance further immune evasion, rather than immune clearance.

### Pomalidomide activates CD4+ T cells without inducing proliferation

**Fig 4:**
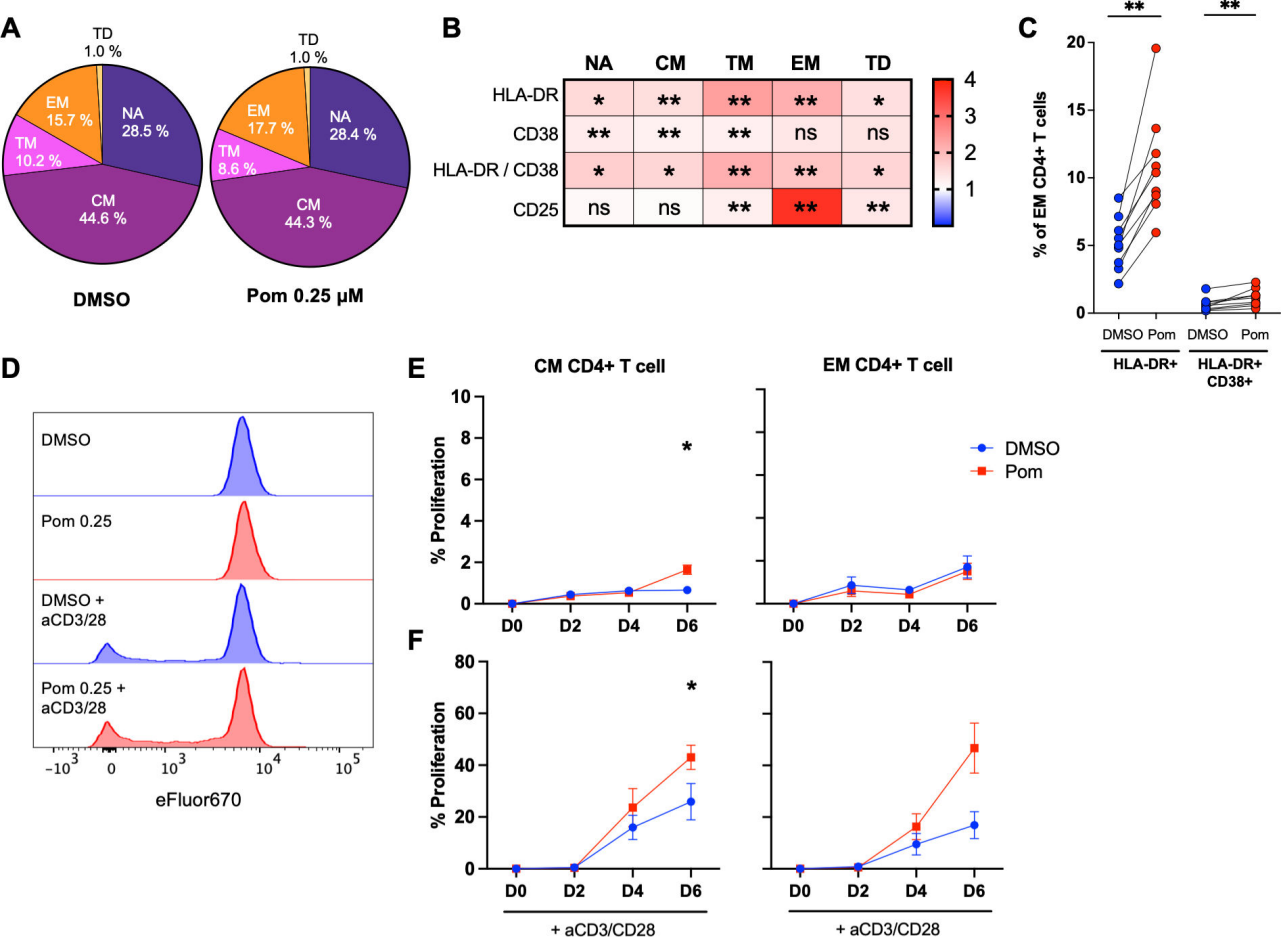
Pomalidomide activates CD4+ T cells without driving proliferation in the absence of TCR stimulation. PBMC from ART-suppressed PLHIV were treated with pomalidomide at 0.25 µM, or DMSO, for 72 hours *ex vivo*. (**A**) The proportion of T cell subsets including NA = naïve, CM = central memory, TM = transitional memory, EM = effector memory, and TD = terminally differentiated is shown (*n* = 9). (**B**) Heatmap showing the median fold change in the expression of activation markers on CD4+ T cell subsets following pomalidomide treatment relative to DMSO control. Red indicates increased expression, and blue indicates reduced expression with pomalidomide treatment relative to the DMSO-treated condition, with the scale of 1–4 representing the fold change. (**C**) Frequency of HLA-DR+ CD38+ and HLA-DR+-expressing cells in the EM CD4+ T cell subset following treatment with DMSO or pomalidomide. (**D**) Representative histogram overlay of the proliferation stain, eFluor670, in DMSO or pomalidomide-treated CD4+ T cells in the absence or presence of T cell receptor (TCR) stimulation with anti-CD3/CD28 antibodies. (**E and F**) Average frequency of proliferating CM and EM CD4+ T cells following DMSO or pomalidomide treatment for 6 days in the (**E**) absence of TCR stimulation or in the (**F**) presence of TCR stimulation. Each symbol in panel C represents a single donor. Each symbol in panels E and F represents the average of six donors. *P < 0.05; **P < 0.01; ns, not significant. Wilcoxon matched-pairs signed-rank test. Bars show median + IQR.

Given previous reports that pomalidomide activates T cells (15), we used a multi-color flow panel to assess the effects of pomalidomide *ex vivo* on peripheral blood mononuclear cells (PBMCs) from PLHIV on suppressive ART. Pomalidomide did not change the distribution of CD4+ T cell memory subsets (Fig. 4A) but induced T cell activation across all CD4+ T cell memory subsets, most notably within EM CD4+ T cells, where compared to DMSO, pomalidomide induced a 2.16- and 1.35-fold-median increase in HLA-DR expression and HLA-DR+ CD38+ expression, respectively (Fig. 4B and C).

As pomalidomide has been described as an antiproliferative agent by upregulating p21 and inducing cell cycle arrest (13) while also stimulating the PI3K/AKT/mTOR pathways to induce proliferation (20–22), we next evaluated the effect of pomalidomide on CD4+ T cell proliferation using cells from PLHIV on ART. We found that pomalidomide induced CD4+ T cell proliferation in CM but had no effect on other T cell subsets (Fig. 4D and E). Knowing that co-stimulatory effects frequently take place in the setting of antigen recognition, we also evaluated if pomalidomide induced proliferation in the setting of TCR stimulation with anti-CD3/CD28. Pomalidomide enhanced TCR-dependent proliferation, with a significant expansion of CM and TM CD4+ T cells after 6 days of pomalidomide treatment (Fig. 4F; Fig. S4A; MFC = 1.5 and MFC = 2.6, respectively). There was a similar increase in EM (MFC = 2.3) in five of the six donors evaluated. The largest proliferating subset following TCR stimulation was CM in both DMSO- and pomalidomide-treated conditions, contributing nearly half of all proliferating CD4+ T cells (Fig. S4B). Taken together, pomalidomide activated CD4+ T cells from PLHIV on ART treated *ex vivo* without inducing proliferation. However, in the presence of TCR co-stimulation, pomalidomide induced further expansion of memory CD4+ T cells.

### Pomalidomide does not reverse HIV latency in CD4+ T cells from PLHIV on ART

**Fig 5:**
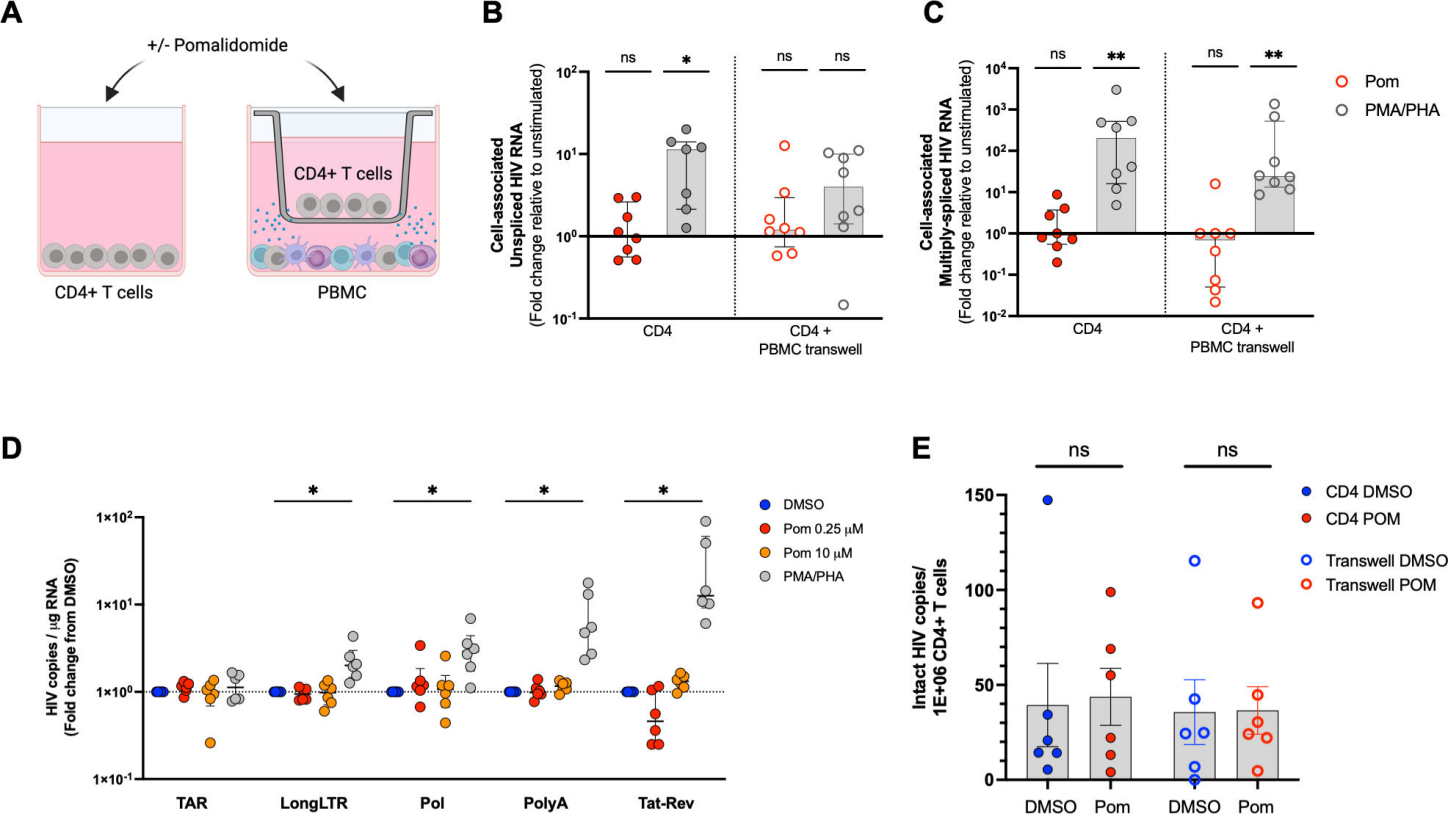
Pomalidomide does not reactivate latent HIV and does not reduce the intact HIV reservoir in CD4+ T cells from ART-suppressed PLHIV. (**A**) CD4+ T cells from ART-suppressed PLHIV were treated with pomalidomide at 0.25 µM, phorbol 12-myristate 13-acetate (PMA)/phytohemagglutinin (PHA) or DMSO, for 72 hours *ex vivo* alone or in a transwell separated from autologous PBMC also exposed to pomalidomide, measuring the fold change induction of (**B**) cell-associated unspliced (US) HIV RNA and (**C**) cell-associated multiply-spliced (MS) HIV RNA relative to the DMSO unstimulated control; (**D**) number of copies of HIV transcripts per 500 ng input RNA, shown as a fold change of drug treated relative to the DMSO vehicle control; and (**E**) frequency of intact HIV DNA in CD4+ T cells following DMSO or pomalidomide treatment for 72 hours. CD4+ T cells were cultured alone (solid circles) or in a transwell membrane separated from autologous PBMC for paracrine stimulation (open circles) in the presence of DMSO or pomalidomide. *P < 0.05; **P < 0.01; ns, not significant. Wilcoxon matched-pairs signed-rank test. Bars show median + IQR.

Given the activation and IC profile of pomalidomide observed at concentrations that can be used therapeutically and recent data suggesting that pomalidomide may activate HIV from latency at higher concentrations (11), we next investigated if pomalidomide induced HIV latency reversal at the therapeutically achievable concentration of 0.25 µM. To do this, CD4+ T cells from PLHIV on ART were treated with pomalidomide or DMSO for 72 hours, and cell-associated unspliced (CA-US) HIV RNA and multiply-spliced (CA-MS) HIV RNA were quantified using qPCR. We found that pomalidomide did not increase CA-US or CA-MS HIV RNA transcripts (Fig. 5A through C; Fig. S5A and B).

As pomalidomide has broad immunomodulatory effects, stimulation of CD4+ T cells by pomalidomide *in vivo* may occur both through direct stimulation and indirectly from paracrine effects from other immune cells responding to pomalidomide. To assess this, CD4+ T cells were separated from autologous PBMC by a transwell membrane, and both were stimulated with pomalidomide (Fig. 5A). Use of the transwell membrane allows for the passage of cytokines and chemokines, enabling the evaluation of paracrine stimulation in addition to direct stimulation of CD4+ T cells on latency reversal, without allowing any effector cell killing of the reactivated HIV-infected CD4+ T cells. Using this experimental model, we found no effect of pomalidomide treatment on HIV latency reversal, with no significant change in CA-US or CA-MS HIV RNA (Fig. 5B and C; Fig. S5A and B).

Given a recent report that pomalidomide at the higher concentration of 10 µM can induce transcription of unspliced HIV RNA in CD4+ T cells from ART-suppressed PLHIV treated *ex vivo* (11) and the potent degradation of Ikaros and Aiolos observed with 10 µM pomalidomide, we also evaluated pomalidomide-induced HIV transcription using both 0.25 and 10 µM concentrations *ex vivo*. We extended the number of HIV transcripts assessed and quantified transcriptional initiation (TAR), elongation beyond the 5′ LTR sequence (long LTR), polymerase (Pol), completion of transcription (PolyA), and multiply-spliced Tat-Rev (Tat-Rev) HIV RNA (47). However, even at the higher 10 µM concentration and two time points of 24 and 72 hours of stimulation, we found minimal effect of pomalidomide on inducing any HIV transcripts in CD4+ T cells from ART-suppressed PLHIV (Fig. 5D; Fig. S5C through E). We did observe a small but significant 2.16-median relative increase in Tat-Rev transcripts with 0.25 µM concentration at 24 hours (Fig. S5C and D), and an observable but non-significant drop in Tat-Rev transcripts at 72 hours (Fig. 5D; Fig. S5E). Taken together, these findings demonstrate that pomalidomide did not substantially induce transcription of latent HIV *ex vivo*.

### Pomalidomide does not reduce the HIV reservoir in CD4+ T cells from PLHIV on ART

A principal component of the “prime, shock and kill” HIV cure strategy involves the combination of a pro-apoptotic agent to prime HIV-infected cells to apoptosis and a latency reversal agent (LRA) to induce HIV protein expression, to ultimately “kill” the reactivated HIV reservoir through viral-induced lysis, apoptosis, and/or immune-mediated clearance. To assess the composite impact of direct exposure of CD4+ T cells to pomalidomide, we quantified changes in the frequency of CD4+ T cells containing intact HIV DNA following pomalidomide *ex vivo* treatment using the intact proviral DNA assay (IPDA) (48, 49). We found that pomalidomide did not alter the frequency of intact HIV DNA in CD4+ T cells, regardless of the absence or presence of paracrine stimulation from PBMC treated with pomalidomide in a transwell system (Fig. 5E). There was also no significant change in the frequency of 5′ defective HIV DNA, but a minor reduction was observed in the hypermutated/3′ deletion (3′ defective) HIV reservoir in CD4+ T cells treated in the absence of PBMC cytokines (Fig. S5F and G ). Collectively, these findings indicate that exposure of isolated CD4+ T cells directly to pomalidomide does not lead to the depletion of cells containing intact HIV.

## DISCUSSION

Various pro-survival mechanisms underlie the persistence of the HIV reservoir in PLHIV on ART. Numerous proteins have been implicated in preventing viral-induced apoptosis, enabling immune escape and/or moderating latency reversal. Ikaros and Aiolos are transcription factors that have been shown to repress HIV transcription and prevent apoptosis (11, 33, 34, 50). Here, we investigated pomalidomide, an immunomodulatory drug that induces degradation of Ikaros and Aiolos, as an agent to sensitize the HIV reservoir to death by lowering the threshold for apoptosis of HIV-infected cells, or through enhancing the detection and clearance of the HIV reservoir by immune effector cells. Using CD4+ T cells from PLHIV on ART, we found that pomalidomide *ex vivo* at a concentration that can be achieved therapeutically activated and induced proliferation of memory CD4+ T cell but did not induce HIV latency reversal or reduce the intact HIV reservoir. Pomalidomide increased the expression of the pro-survival factor Bcl-2 and the stress proteins ULBP-2/5/6 in HIV productively infected CD4+ T cells, but this did not sensitize HIV productively infected CD4+ T cells to NKG2D-mediated NK cell killing. Contrary to a previous published study (11), we found that pomalidomide did not induce latency reversal in latently infected CD4+ T cells.

Our study represents the most comprehensive analysis of the direct effects of pomalidomide on HIV-infected CD4+ T cells from PLHIV on suppressive ART. Previous studies evaluated the HIV latency reversal and pro-apoptotic effects of pomalidomide using HIV-infected cell lines (29) or in isolated CD4+ T cells but at concentrations much higher than what can be achieved clinically (11). In prior clinical studies in PLHIV receiving pomalidomide for the management of a specific malignancy, such as Kaposi’s Sarcoma, the effects on the HIV reservoir and viral reactivation were not evaluated (15, 51–53). Interestingly, in a recent clinical trial for HIV-associated cryptococcal meningitis in PLHIV on ART, evaluating another thalidomide derivative, lenalidomide, the investigators describe a reduction in cell-associated HIV RNA (54).

Pomalidomide has been shown to induce viral expression in lytic EBV latent Burkitts lymphoma cell line models and HTLV-I macaque models (21, 55), and more recently shown to reverse HIV latency in isolated CD4+ T cells from three virally suppressed PLHIV; however, this required a very high concentration of 10 µM, which is unachievable with clinical dosing, and latency reversal was not seen at 1 µM (11). In our study, we evaluated pomalidomide at 0.25 µM, consistent with the C_MAX_ obtained with daily dosing of 5 mg in a clinical trial for Kaposi Sarcoma, which was well tolerated (30). We also evaluated the effects on reversing latency using 72 hours of exposure to pomalidomide to align with the conditions that consistently induced CD4+ T cell activation, and to allow enough time for autocrine and paracrine stimulation. Even when employing conditions used in a previous study, with CD4+ T cells isolated from ART-suppressed PLHIV exposed to pomalidomide for 16–24 hours (11), or conditions that more closely recapitulate physiological and pharmacological *in vivo* dynamics, pomalidomide did not enhance latency reversal. Differences between this and the previous study include a greater number of donors evaluated in our investigation (six to eight participants compared to three, respectively) and participants were on ART for longer in this study compared to the previous study (median time of 240 and 85 months, respectively). We also measured latency reversal by cell-associated unspliced and multiply-spliced HIV-1 RNA at 24 and 72 hours, compared to the previous study that measured latency reversal by detection of HIV-1 gag RNA at 16 hours (11). The inability of pomalidomide to reverse HIV latency may reflect a number of blocks to transcription that persists despite Ikaros and Aiolos degradation, such as persisting Ikaros-induced polycomb repressive complex 2 (PRC2)-mediated chromatin repression, or insufficient activation of PI3K and Akt to support FOXO1 knockdown and subsequent mTOR activation, impeding activating transcription factor-4 (ATF4)- or nuclear factor of activated T cell (NFAT)-mediated HIV transcription (17, 22, 55–57).

Given previous data consistent with apoptosis-promoting effects from pomalidomide (26, 27, 58), we investigated how pomalidomide may impact cellular apoptosis in HIV-infected and uninfected cells. We did not observe pro-apoptotic effects similar to what was previously described in cancer cell lines (26, 27), with no elevation in active/cleaved caspase-8 or other markers of apoptosis. A potential explanation for this discrepancy is different expression levels and epigenetic modifications controlling the expression of cereblon, Ikaros, and Aiolos, between cancer cell lines and primary cells, as well as differing downstream IL-2-mediated effects following pomalidomide treatment (59). Aiolos expression in various cell lines is predominately under the control of DNA methylation, whereas in hematopoietic primary cells, such as T cells, histone modifications have been shown to predominately control Aiolos expression (59). IL-2 has previously been shown to protect against activation-induced cell death (AICD) through the Jak/STAT/PI3K activation cascade, simultaneously supporting T cell activation and differentiation, and elevating the expression of the pro-survival proteins Bcl-2 and X-linked IAP (XIAP) in conventional T cells (60, 61). Hence, while pomalidomide is described as pro-apoptotic through caspase-8 upregulation and inhibiting IAP-2 in cancer cell lines (26, 27), in this study, we observed that pomalidomide treatment paradoxically supported cell survival through upregulating Bcl-2, improving the viability of CD4+ T cells. Furthermore, the greatest effects of pomalidomide-mediated Bcl-2 upregulation were seen in productive HIV-infected cells. It should be noted that multiple pro-apoptotic and anti-apoptotic proteins play a role in regulating programmed cell death, and we did not extensively investigate all proteins in this study. With this caveat in mind, combined with the lack of latency reversal induced by pomalidomide, these data support our key finding that exposure of pomalidomide to CD4+ T cells does not directly induce lysis or apoptosis in HIV-infected cells.

Previous studies have also shown pomalidomide to confer antiproliferative effects through impairing c-Myc, a master regulator of proliferation, and upregulating p21^WAF-1^, a cell cycle inhibitor (23–25), while IL-2 stimulation of the PI3K/AKT/mTOR pathways may induce proliferation (20–22). We, therefore, assessed whether pomalidomide increased clonal expansion of the HIV reservoir given its effects on IL-2-driven proliferation (23–25, 62). This feature was of high interest, given recent evidence that Aiolos, the transcription factor degraded following pomalidomide treatment, promotes proliferation of both latent and productive HIV-infected CD4+ T cells in PLHIV on ART, and that both latent and transcriptionally active HIV-infected cells display significantly elevated expression levels of Aiolos and higher expression of the proliferation marker Ki67 (10). We found enhanced proliferation by pomalidomide, only when combined with a potent TCR stimulus. This likely reflects synergistic activation of the PI3K/AKT/mTOR pathway to induce proliferation (20–22). Immunomodulatory imide drugs have been shown previously to synergize with TCR/antigen-dependent signaling to induce T cell activation (63–66). Notably, in our experiments, pomalidomide increased levels of CD4+ T cell activation even in the absence of TCR/antigen co-stimulation, which could be due to persisting immune activation in PLHIV on ART (67, 68) that differs from an HIV-negative donor.

A notable finding from our study was that pomalidomide elevated stress protein expression, namely ULBP-2,5,6, on productive HIV-infected cells, which could potentially support immune clearance of a productively infected cell. Indeed, in a mouse model, T cells treated with IL-2 were eliminated by stimulated NK cells in a perforin-dependent manner, mediated through NKG2D–stress protein interactions (69). While we did not observe a reduction in CD4+ T cells when cultured with the NK cell line, KHYG-1, the NK cell line may not fully recapitulate the complex interaction between NK cells and CD4+ T cells, and thus requires further evaluation.

Interestingly, we also observed a significant downregulation of the immune checkpoint markers TIGIT and TIM-3, as well as a downregulation of the immune senescence marker, CD57, in EM CD4+ T cells in samples from PLHIV. TIGIT has been implicated in supporting HIV persistence through moderating viral reactivation and supporting immune evasion (5, 6). There is conflicting information on the role of TIM-3 and HIV persistence, with TIM-3 expression correlated with viral load but also shown to facilitate activation and cytokine production in human T cells *in vitro* (70, 71). The observed downregulation of TIGIT and TIM-3 on EM CD4+ T cells has not been previously described with pomalidomide treatment nor IL-2 therapy. Notably, these changes were accompanied by a significant upregulation of PD-L1 and CD155 on EM CD4+ T cells, which could be secondary to the increased IL-2 production (72). These changes are of interest, given the expression of their corresponding inhibitory receptors, PD-1 and TIGIT, respectively, is elevated on NK cells and HIV-specific CD8+ T cells in PLHIV on ART, and thus may facilitate immune escape (73–76). An important caveat to our findings is that we measured immune checkpoint markers and CD155 in samples from PLHIV without discriminating between infected and uninfected CD4+ T cells, so we were unable to track effects that may be specific to CD4+ T cells harboring HIV.

We recognize several limitations of our study that require further consideration. First, it is worth acknowledging that our investigations were restricted to male PLHIV, recruited in Australia, the USA, and Denmark, and predominately infected with clade B virus. Second, intact HIV DNA quantification was restricted to donors with reservoirs detectable with our IPDA clade B primer/probe sets. Third, the evaluation of pomalidomide on CD4+ T cell responses and the HIV reservoir were on CD4+ T cells isolated from the peripheral blood and may not reflect the effect of pomalidomide on the reservoir that persists in other anatomical sites. Fourth, it has been described that Aiolos is upregulated in latent and productive proliferating HIV reservoir (10); however, whether Aiolos expression is altered with longer ART suppression has not been characterized and may alter the sensitivity of the HIV reservoir to the effects of pomalidomide treatment. Finally, we evaluated the effect of pomalidomide on MHC-I, stress proteins, and other key receptors for immune clearance in productive infection model systems, which may not reflect the effects of pomalidomide on latently infected cells or latently infected cells that were recently activated. We used an EGFP reporter virus model to enable efficient and sensitive detection of the effect of pomalidomide on HIV productively infected cells (GFP+) relative to GFP–, which are largely HIV uninfected but may also contain non-productively infected and abortively infected cells. The use of the GFP reporter virus also enabled the profiling of a greater frequency of HIV productively infected cells compared to CD4+ T cells from PLHIV. The limitation of this model was that the CD4+ T cells were stimulated to enhance infection with the R5-tropic, EGFP-reporter virus, and while the CD4+ T cells were rested for 48 hours, there may be persisting immune activation. In addition, productively infected cells may not fully recapitulate the survival and proliferative capacity of latently infected cells or latently infected cells that have recently reactivated.

In conclusion, we found that targeting Ikaros and Aiolos in CD4+ T cells with pomalidomide did not promote latency reversal, apoptosis, and viral-induced cytolysis of the HIV reservoir or sensitize HIV-infected cells to NK cell killing. Pomalidomide was found to induce the expression of Bcl-2, which could protect HIV-infected cells from apoptosis and immune-mediated clearance. We conclude that targeting Ikaros and Aiolos in CD4+ T cells is unlikely to contribute to viral reactivation or directly induce apoptosis of the HIV reservoir.

## MATERIALS AND METHODS

### Experimental model and subject design

PBMCs were isolated by Ficoll-Paque density gradient centrifugation (Cytiva; 17144003) from buffy coats obtained from the Australian Red Cross Lifeblood (Melbourne, Australia). PBMCs were treated with ACK lysing buffer (Gibco, A10492-01) to remove erythrocytes and rested overnight at 2e+06 cells/mL before culturing. PBMCs were cultured at 1e+06 cells/mL in assay conditions. Large numbers of PBMCs were collected from PLHIV on suppressive ART by leukapheresis at the Alfred Hospital, Melbourne, Australia, after obtaining informed consent with approval from the relevant Institutional Review Boards (Alfred Hospital, Melbourne, Australia, HREC 214/15 and 48/16; The Avenue Hospital, Windsor, Australia, HREC 00242, protocol 202; The University of Melbourne, Australia, HREC 15452271 and 1443071). All study participants were male. Plasma HIV RNA was below the detection limit of the utilized assay (<20 or <40 copies/mL) at the time of leukapheresis. Median duration of suppressed plasma HIV RNA was 10.7 years (interquartile range [IQR] = 8.25–12.75), and median nadir was 230 cells/µL (IQR = 76.0–319.5). Additional patient demographics and ART regimen are listed in Table S1.

Following leukapheresis, PBMCs from PLHIV were isolated by Ficoll-Paque density gradient centrifugation, resuspended in fetal bovine serum (FBS) with 10% DMSO, and stored in liquid nitrogen. Upon thawing, PBMCs were rested overnight in the presence of HIV integrase inhibitor, raltegravir (1 µM; Selleck Chem; S2005) in RPMI 1640 medium (Life Technologies; 11875093) supplemented with 10% (vol/vol) heat-inactivated FBS, 100 U/mL penicillin, 100 µg/mL streptomycin, and 292 µg/mL glutamine (Gibco; 10378016) (RF-10). PBMCs were then cultured with the appropriate drug conditions at 1–1.5e+06 cells/mL in the presence of ART (raltegravir). Pomalidomide (Sigma-Aldrich; P0018) was assessed in PBMC culture in RF-10 medium at the therapeutically relevant concentration of 0.25 µM, which corresponds to the C_MAX_ of *in vivo* pomalidomide administration (30), or 10 µM where appropriate, and compared to a DMSO vehicle control.

### Cell lines

HEK 293T (obtained from NIH) and TZM-bl (obtained from NIH) cell lines were grown as a monolayer in Dulbecco’s minimal essential medium (DMEM; Gibco) supplemented with 10% (vol/vol) heat-inactivated FBS, 100 U/mL penicillin, 100 µg/mL streptomycin, and 292 µg/mL glutamine (Gibco) at 37°C with 5% CO_2_. KHYG-1 (obtained from A. Barrow, The University of Melbourne, Australia) was cultured in RF-10 with 100 U/mL interleukin-2 (IL-2; R&D Systems; RDS202IL500) at 37°C and 5% CO_2_.

### Western blots

Total CD4+ T cells from PLHIV on suppressive ART were negatively selected using magnetic cell sorting with EasySep Human CD4+ T cell Isolation Kit (StemCell Technologies; 17952) and were rested overnight before culturing with pomalidomide (0.25, 2, 10 µM) or DMSO for 24 and 72 hours. CD4+ T cells were harvested after drug treatment and were lysed with lysis buffer containing 50 mM Tris, 150 mM NaCl, 1.0% NP40, 1% sodium deoxycholate, 0.1% SDS, HCl pH 7.6, Halt protease and phosphatase inhibitor cocktail (Thermo Scientific; 78440), and benzonase endonuclease (Merck; E1014). Lysate protein concentrations were normalized based on protein quantification using the Pierce Bradford Protein Assay Kit (Invitrogen; 23200), and lysates were mixed with Laemmli buffer (Bio-Rad; 1610747) and 1 M DTT (Sigma; 43816) and were heated for 5 min at 95°C. The samples were run on a Mini-PROTEAN TGX Stain-Free Gel 4%–15% (Bio-Rad; 456-8083) and transferred to a polyvinylidene fluoride (PVDF) membrane (Bio-Rad; 1704156). The membrane was blocked with 5% skimmed milk solution, with Ikaros (IKZF1) and Aiolos (IKZF3) separately stained and detected using the anti-IKZF1 polyclonal antibody (Invitrogen; PA5-85570) and the anti-IKZF3 polyclonal antibody (Invitrogen; 720418). Primary antibodies were followed by a secondary horseradish peroxidase (HRP)-conjugated mouse anti-rabbit antibody (GenScript; A01827). Membranes were visualized on the Bio-Rad ChemiDoc machine using the chemiluminescence detection system. GAPDH was visualized on the blots following stripping with the Restore Western blot stripping buffer (Thermo Fisher; 21059) and was blocked with a 5% skimmed milk solution. GAPDH was measured following staining with the anti-GAPDH polyclonal antibody (Invitrogen; PA1-987) and the secondary HRP-conjugated mouse anti-rabbit antibody, as described and visualized with the chemiluminescence detection system.

### Virus and infection

Plasmid DNA encoding an NL4.3 backbone with an AD8 envelope and enhanced green fluorescent protein (EGFP) inserted one base downstream of the env open reading frame, followed by an IRES-Nef coding sequence, kindly provided by Damian Purcell (University of Melbourne) and Yasuko Tsunetsugu-Yokota (National Institute of Infectious Diseases, Tokyo, Japan), was transiently transfected into HEK 293T cells using FuGENE (Promega; E2692). Cells were washed 24 hours after transfection, and the culture continued for an additional 24 hours. Virus supernatant was harvested 2 days after transfection, filtered by passage through 0.45 µM filter and concentrated using Lenti-X Concentrator (Takara Bio; 631232) per manufacturer’s instructions, and stored in aliquots at −80°C. TCID50 of the full-length, Nef-competent EGFP-reporter virus was determined on TZM-bl cells using the Reed and Muench method (77), and read using luciferase as a read-out, as described previously (Promega; E1501) (78).

### HIV infection model

Total CD4+ T cells were negatively selected using magnetic cell sorting with EasySep Human CD4+ T cell Isolation Kit. CD4+ T cells were stimulated with Phytohemagglutinin (PHA; 20 µg/mL; Thermo Fisher; R30852701) and IL-2 (10 U/mL; R&D Systems; RDS202IL500) for 48 hours. After 48 hours of stimulation, the stimulated CD4+ T cells were infected with full-length, Nef-competent EGFP-reporter virus at TCID50 = 1.0 for 2–3 hours, washed, and continued in culture with IL-2 (10 U/mL) for 48 hours.

### Quantification of apoptosis

Forty-eight hours post-infection, the *in vitro* infected CD4+ T cells were treated with pomalidomide (0.25 µM), DMSO, or camptothecin (Sigma-Aldrich; C9911) as a positive control for apoptosis. CD4+ T cells were harvested after drug treatment for 2, 4, and 6 days. Cells were counted using the Countess 3 Automatic cell counter (Thermo Fisher). Apoptotic cells were measured by staining with Live/Dead Fixable Near Infra-Red (NIR) Viability Stain (Invitrogen; L34975) and anti-Annexin-V-BUV395 (BD Biosciences; 564841) with Annexin Binding Buffer (Invitrogen; V13246). Caspase activation was measured using Image-iT LIVE Red Caspase Detection Kit (Invitrogen; I35102) and Red Active Caspase 8 Staining Kit (Abcam; AB273416). The anti-apoptotic protein, Bcl-2, was measured using anti-Bcl-2-PE (BioLegend; clone 100), following permeabilization of cells using BD Cytofix/Cytoperm 1× solution (BD; 544714). Cells were fixed with 1% paraformaldehyde, and acquisitions were performed on the BD LSRFortessa. Flow cytometry analyses were conducted using OMIQ software (Dotmatics). Antibodies to surface markers CD3 and CD4 were not included, as dead cells have been shown to non-specifically bind antibodies, and dead, apoptotic, and live cells were of key interest for this profiling. High CD4+ T cells purity (>95%) by StemCell isolation enabled direct CD4+ T cell profiling, using the following gating strategy: exclusion of debris > single cells > Live/Dead/Annexin V > GFP > caspase or Bcl-2.

### Stress ligands and NK cell ligands investigation on HIV-infected CD4+ T cells

Using the *in vitro* HIV productive infection assay, *in vitro* infected CD4+ T cells were treated with DMSO, pomalidomide (0.25 µM), or Staphylococcal enterotoxin B (SEB) (Sigma-Aldrich; S4881; 1 µg/mL) as a positive control, with a media change after 72 hours of culturing with drug treatments. Measurements were performed after drug treatment for 24 hours, 3 days, and 5 days. Cells were stained with Live/Dead Fixable Blue Viability Stain (Invitrogen; L23105) and with the following antibodies: anti-CD3-APC Fire750 (BioLegend; clone UCHT1), anti-CD4-BV785 (BioLegend; clone RPA-T4), anti-CD155/PVR-PE (BD; clone SKII.4), anti-HLA-(A,B,C)-APC (BioLegend; clone W6/32), anti-HLA-E-PE (BioLegend; clone 3D12), anti-MIC(A/B)-PE/Cy7 (BioLegend; clone 6D4), and anti-ULBP-(2,5,6)-BV650 (BD; clone 165903). Staining for CD155 was performed separately from the staining for HLA-E. Cells were fixed with 1% paraformaldehyde, and acquisitions were performed on the Cytek Aurora. Flow cytometry analyses were conducted using OMIQ software. As productive HIV infection is associated with the downregulation of the CD3 and CD4 receptors (79, 80), the flow gating strategy for CD4+ T cells was single cells > live > CD3^dim+high^ > CD4^dim+high^.

### KHYG-1 killing assays

*In vitro*-infected CD4+ T cells treated with DMSO or pomalidomide (0.25 µM) were pre-stained with Cell Proliferation Dye eFluor670 (Invitrogen; 65-0840-90), per the manufacturer’s recommendations. CD4+ T cells were co-cultured with the KHYG-1 NK cell line (obtained from A. Barrow) at the E:T ratio of 1:1 for 4 hours. Cells were then stained with Live/Dead Fixable-Violet Dead Cell Stain (Invitrogen; L34955), with the frequency of GFP+ CD4+ T cells quantified to measure a relative change in the frequency of HIV-infected cells following co-culture. The percentage of killing (%killing) of target cells by KHYG-1 cells was calculated using the following formula: [(GFP^+^ cells in targets) − (%GFP^+^ cells in targets + effectors)] / (%GFP^+^ cells in targets) × 100.

To investigate the role of NKG2D in interactions crucial for killing, conditions were treated with or without an NKG2D-blocking antibody (BioLegend; clone 1D11). KHYG-1 cells were pre-incubated with the anti-NKG2D antibody for 30 minutes prior to co-culture (10 µg/mL). The percentage of killing (%killing) of target cells by KHYG-1 cells was calculated using the following formula: [(GFP^+^ cells in targets + anti-NKG2D antibody) − (%GFP^+^ cells in targets + effectors + anti-NKG2D antibody)] / (%GFP^+^ cells in targets + anti-NKG2D antibody) × 100.

### *Ex vivo* phenotyping studies of PBMC from people living with HIV

Phenotypes of PBMC from PLHIV treated *ex vivo* with pomalidomide were assessed in a large flow panel using the following antibodies: anti-CD3-BV750 (BioLegend; clone SK7), anti-CD4-PE Cy7 (BioLegend; clone OKT4), anti-CD8-PerCP-Cy5.5 (BioLegend; clone SK1), anti-CD45RA-AF488 (BioLegend; clone HI100), anti-CCR7-AF700 (BioLegend; clone G043H7), anti-CD27-BV785 (BioLegend; clone O323), anti-CD25-APC-Fire750 (BioLegend; clone BC96), anti-HLA-DR-BV570 (BioLegend; clone L243), anti-CD38-BV510 (BioLegend; clone HIT2), anti-PD-1-BUV661 (BD; clone EH12.1), anti-TIGIT-BV605 (BioLegend; clone A15153G), anti-TIM-3-Superbright436 (Invitrogen; clone F38-2E2), anti-PD-L1-BUV496 (BD; clone MIH1), anti-CD57-eFluor450 (Invitrogen; clone TB01), anti-CD56-PerCP-eFluor710 (Invitrogen; clone TULY56), anti-CD16-PE/Dazzle594 (BioLegend; clone 3G8), anti-NKG2A-APC (Miltenyi-Biotec; clone REA110), anti-NKG2C-BV480 (BD; clone 134591), anti-NKG2D-BB515 (BD; clone 1DII), anti-NKp30-BUV805 (BD; clone p30-15), anti-NKp44-BUV395 (BD; clone p44-8), anti-DNAM-1(CD226)-BUV563 (BD; clone DX11), anti-CD14-BV421 (BD; clone Mϕp9), anti-CD40-BV711 (BioLegend; clone 5C3), anti-CD86-AF647 (BioLegend; clone IT2.2), anti-CD123-PE/Cy5 (BioLegend; clone 6H6), anti-CD11c-BUV737 (BD; clone B-ly6), anti-CD155-PE (BD; clone SKII.4), anti-CD19-AF532 (Invitrogen; clone SJ25C1), and Zombie NIR Fixable Viability Kit (BioLegend; 423106). StemPro Accutase Cell Dissociation Reagent (Invitrogen; A1110501) was used to detach monocytes and dendritic cells from the well. Following staining, cells were fixed with 1% paraformaldehyde, and acquisitions were performed on the Cytek Aurora. Flow cytometry analyses were conducted using OMIQ software.

CD4+ T cell subsets were defined as follows: naïve (NA) = CD3+CD4+CD45RA+CCR7+, central memory (CM) = CD3+CD4+CD45RA-CCR7+CD27+, transitional memory (TM) = CD3+CD4+CD45RA-CCR7-CD27+, effector memory (EM) = CD3+CD4+CD45RA-CCR7-CD27-, and terminally differentiated (TD) = CD3+CD4+CD45RA+CCR7-. Gating was performed on fluorescence minus one (FMO) controls.

### Activation and proliferation assays

PBMCs from PLHIV were labeled with CellTrace Violet Proliferation Stain (CTV; Invitrogen; C34557) according to the manufacturer’s instructions. On day 1, PBMCs were cultured with drug treatments at 1e+06 cells/mL, in the absence of stimulation or in plates pre-coated with purified anti-CD3 (2 µg/mL; BioLegend; clone OKT3) with soluble, purified anti-CD28 (0.1 µg/mL; BioLegend; clone CD28.2). Measurements were taken on days 3, 5, and 7, with a half-media change performed every 72 hours. Staining was performed with the following antibodies: anti-CD3-PE (BD; clone UCHT1), anti-CD4-PE Cy7, anti-CD8-BUV805 (BD; clone SK1), anti-CD45RA-PerCP-Cy5.5 (BioLegend; clone HI100), anti-CCR7-AF700, anti-CD27-BV785, anti-HLA-DR-BV570, anti-PD-1-BUV661, and Zombie NIR Viability Stain. Cells were fixed with 1% paraformaldehyde, and acquisitions were performed on the Cytek Aurora. Flow cytometry analyses were conducted using FlowJo.

### Reversal of HIV latency

PBMCs from PLHIV were sorted into CD4+ T cells using the EasySep Human CD4+ T cell Isolation Kit and were rested overnight. Unsorted PBMCs were also rested overnight. CD4+ T cells were then cultured with pomalidomide (0.25 µM), Phorbol 12-Myristate 13-Acetate (PMA; 20 nM; Sigma-Aldrich; P1585) and PHA (20 µg/mL), or DMSO vehicle control at 2e+06 cells/mL in RPMI-supplemented media with raltegravir (1 µM). To assess the role of pomalidomide-induced cytokines from PBMC in stimulating CD4+ T cell latency reversal, PBMCs were co-cultured with CD4+ T cells at 1:1, with CD4+ T cells cultured in a 0.4 µM pore insert transwell membrane (Corning; CLS3460) to allow cytokine diffusion between PBMCs and CD4+ T cells. Cells were cultured at a final 2e+06 cells/mL. CD4+ T cells were collected after 72 hours of drug treatment, lysed using a Qiashredder kit (Qiagen; 79654), and stored at −80°C.

To quantify levels of cell-associated (CA-)HIV RNA and DNA, RNA and DNA were extracted from lysates using the Qiagen AllPrep DNA/RNA Mini Kit (Qiagen; 80204). CA-unspliced (US) HIV RNA was quantified using a semi-nested real-time quantitative polymerase chain reaction (RT-PCR) as previously described (81–83). CA-multiply-spliced (MS) HIV RNA was measured using RT-PCR as described previously (81). HIV RNA copy numbers were interpolated from the standard curve and expressed normalized to nanodrop input rather than 18S rRNA quantification. For data measurements that were detectable but less than the single copy standard, 0.5 was designated as the HIV RNA copy number. Due to the limitations of the assay, measurements that had zero-detectable HIV RNA were designated 0.1 HIV RNA copies for fold change calculations.

### HIV RNA transcriptional profiling

PBMCs from PLHIV were sorted into CD4+ T cells using the EasySep Human CD4+ T cell Isolation Kit and were rested overnight. CD4+ T cells were then cultured with pomalidomide (0.25, 10 µM), PMA (10 nM) and PHA (10 µg/mL), or DMSO vehicle control at 2e+06 cells/mL in RPMI-supplemented media with raltegravir (1 µM). CD4+ T cells were collected after 24 and 72 hours of drug treatment, and were stored at −80°C. RNA was isolated using TRI Reagent (Thermo Fisher; AM9738) according to the manufacturer’s recommendations. HIV RNA assays measuring TAR, long LTR, Pol, PolyA, and Tat-Rev were measured and analyzed as described (47). Due to the limitations of the assay, measurements that had zero-detectable transcripts were designated 0.1 RNA copies for fold change calculations.

### HIV reservoir quantification

To quantify levels of intact HIV DNA, the Intact Proviral DNA Assay (IPDA) was performed as described using the QIAcuity Four 5plex System (48, 49), with primers and probes listed in Table S2.

### Data analysis and statistics

Data were examined for normal distribution using GraphPad Prism’s normality algorithm, and visual inspection of frequency histograms and quantile–quantile plots. Parametric or non-parametric tests were then applied as appropriate with statistical comparisons between experimental conditions performed by paired *t*-test or Wilcoxon signed-rank test.

## Data Availability

This paper does not report original code. The data sets reported in this paper are available from the corresponding author upon request.

## SUPPLEMENTAL MATERIAL

Figure S1 jvi.01676-24-s0001.tiff Pomalidomide degrades Ikaros and Aiolos in CD4+ T cells from PLHIV on suppressive ART after 24 and 72 hours treatment *ex vivo*.

Figure S2 jvi.01676-24-s0002.tiff Pomalidomide upregulates stress protein expression on HIV-infected and HIV-uninfected CD4+ T cells.

Figure S3 jvi.01676-24-s0003.tiff Pomalidomide elevates CD155 and PD-L1, and reduces TIGIT expression on memory CD4+ T cells from ART-suppressed PLHIV.

Figure S4 jvi.01676-24-s0004.tiff In the presence of stimulation, pomalidomide enhances the proliferation of memory CD4+ T cells.

Figure S5 jvi.01676-24-s0005.tiff Pomalidomide does not induce latency reversal in CD4+ T cells from ART-suppressed PLHIV.

Supplemental legends and tables jvi.01676-24-s0006.docx Legends for Fig. S1 to S5; Tables S1 and S2.

ASM does not own the copyrights to Supplemental Material that may be linked to, or accessed through, an article. The authors have granted ASM a non-exclusive, world-wide license to publish the Supplemental Material files. Please contact the corresponding author directly for reuse.
