# Supplementary figures and images for: Targeting Ikaros and Aiolos with pomalidomide fails to reactivate or induce apoptosis of the latent HIV reservoir
Source: J Virol. 2025 Feb 4;99(3):e01676-24. doi: 10.1128/jvi.01676-24 (PMC11915836; doi:10.1128/jvi.01676-24)

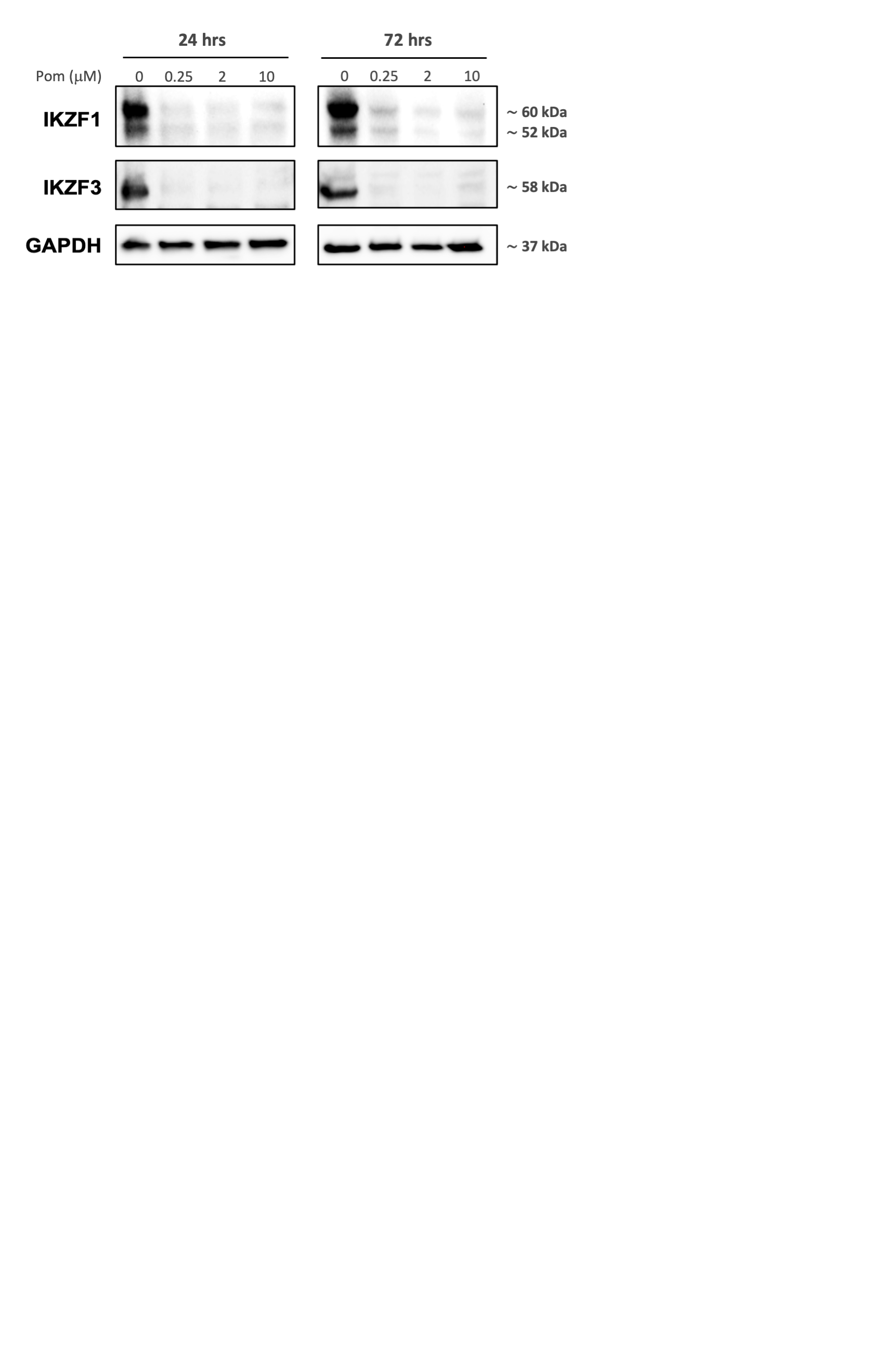

Supplement: Figure S1 — Pomalidomide degrades Ikaros and Aiolos in CD4+ T cells from PLHIV on suppressive ART after 24 and 72 hours treatment ex vivo. [file jvi.01676-24-s0001.tiff]

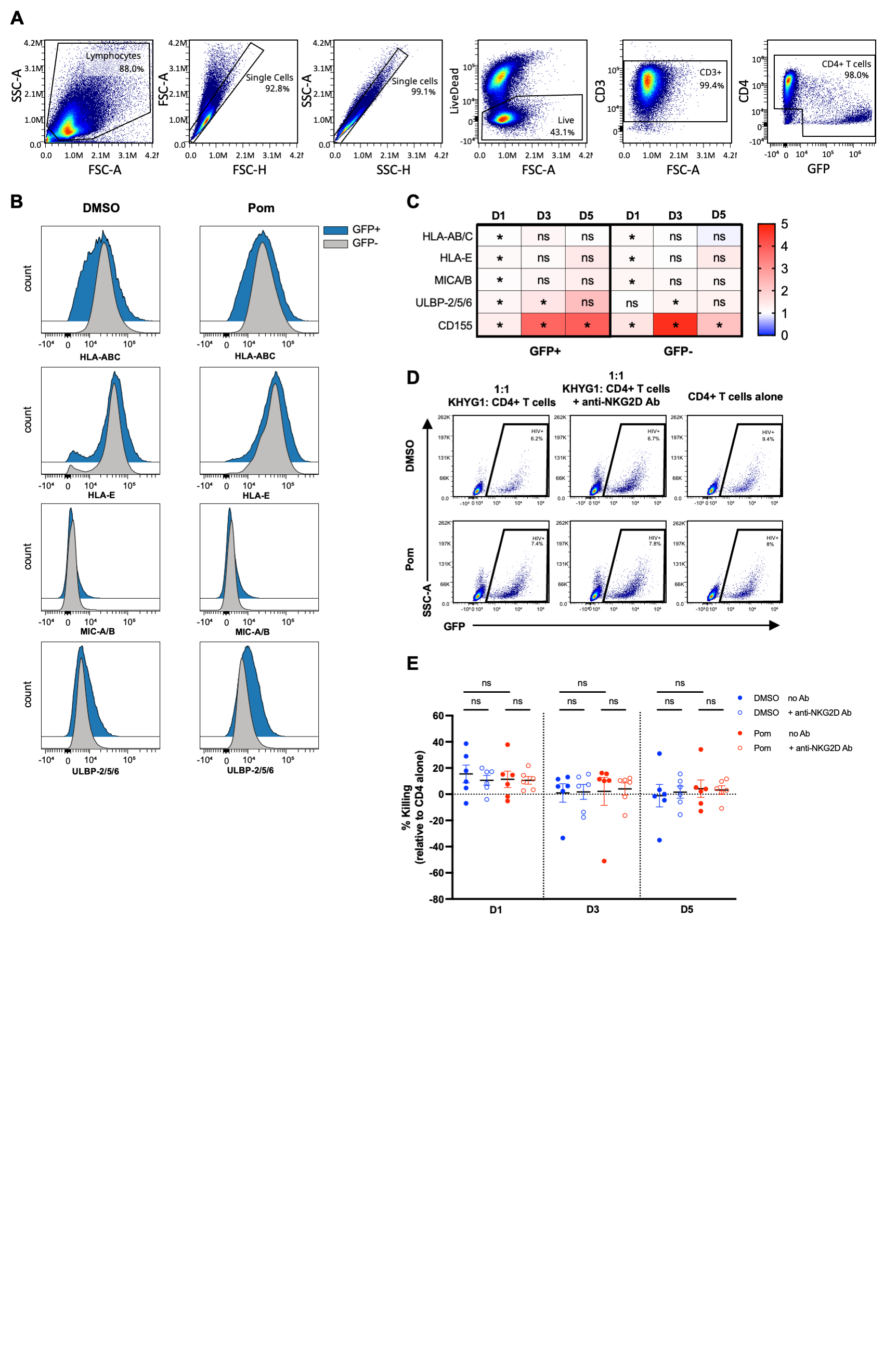

Supplement: Figure S2 — Pomalidomide upregulates stress protein expression on HIV-infected and HIV-uninfected CD4+ T cells. [file jvi.01676-24-s0002.tiff]

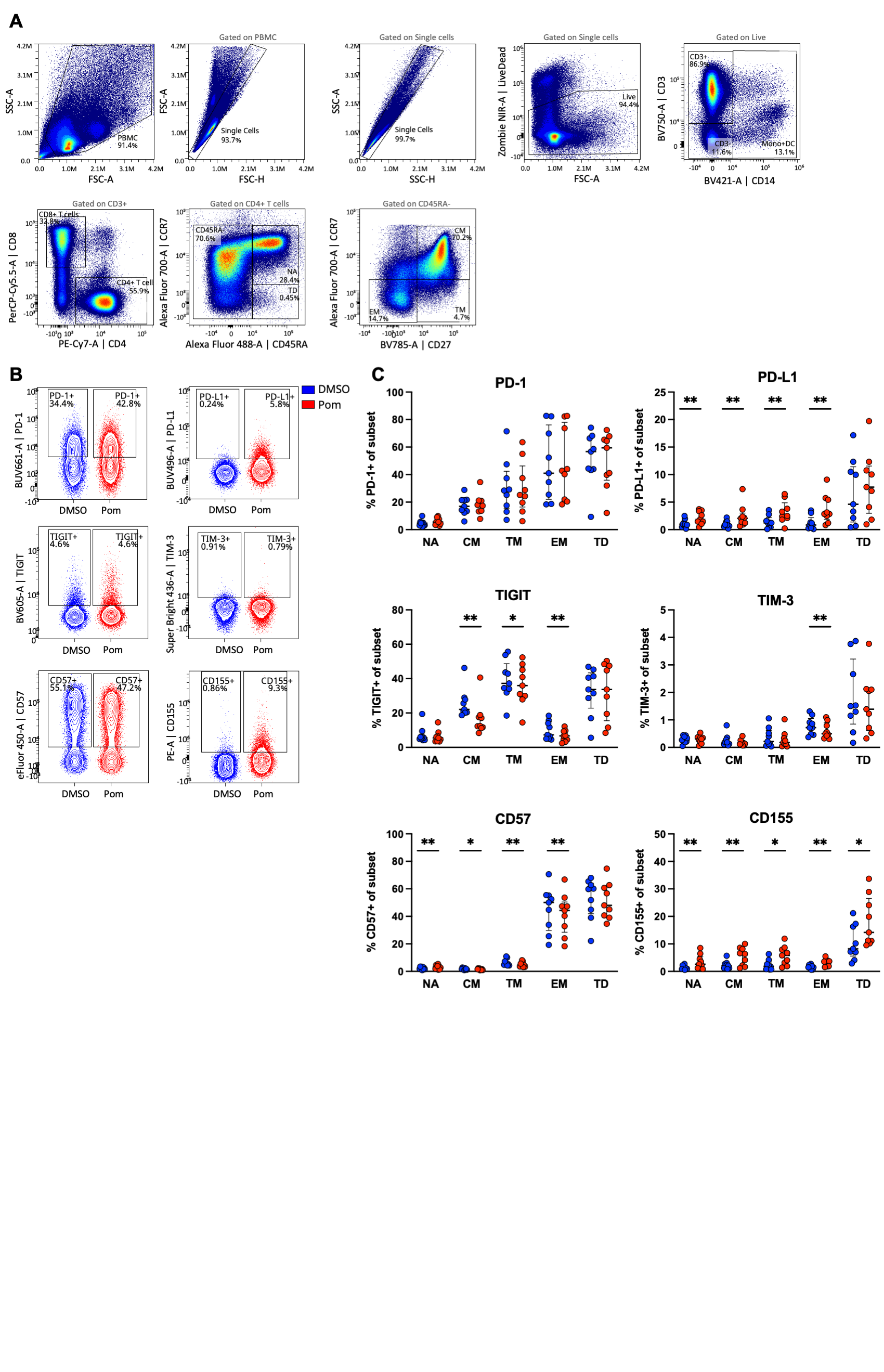

Supplement: Figure S3 — Pomalidomide elevates CD155 and PD-L1, and reduces TIGIT expression on memory CD4+ T cells from ART-suppressed PLHIV. [file jvi.01676-24-s0003.tiff]

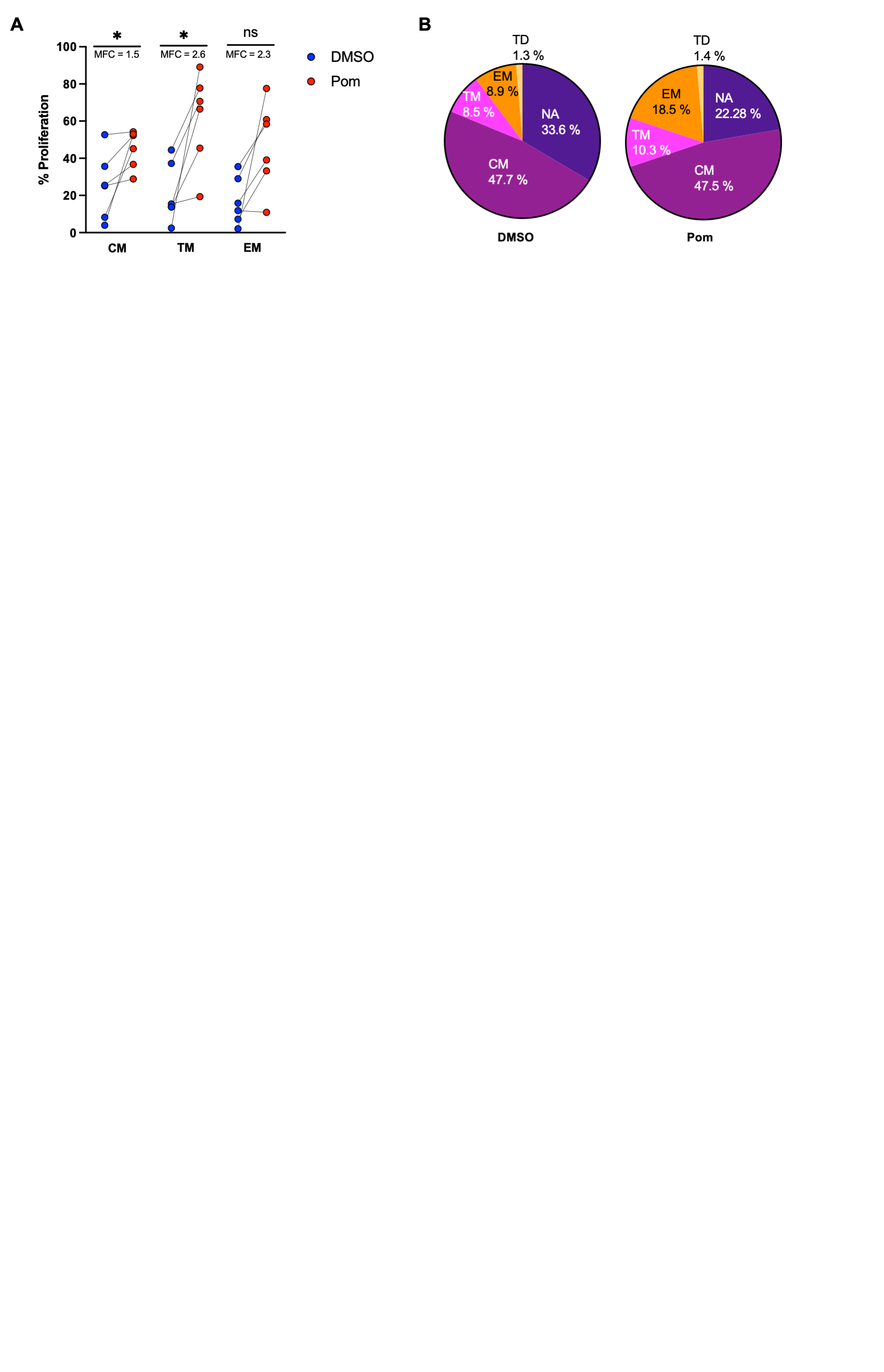

Supplement: Figure S4 — In the presence of stimulation, pomalidomide enhances the proliferation of memory CD4+ T cells. [file jvi.01676-24-s0004.tiff]

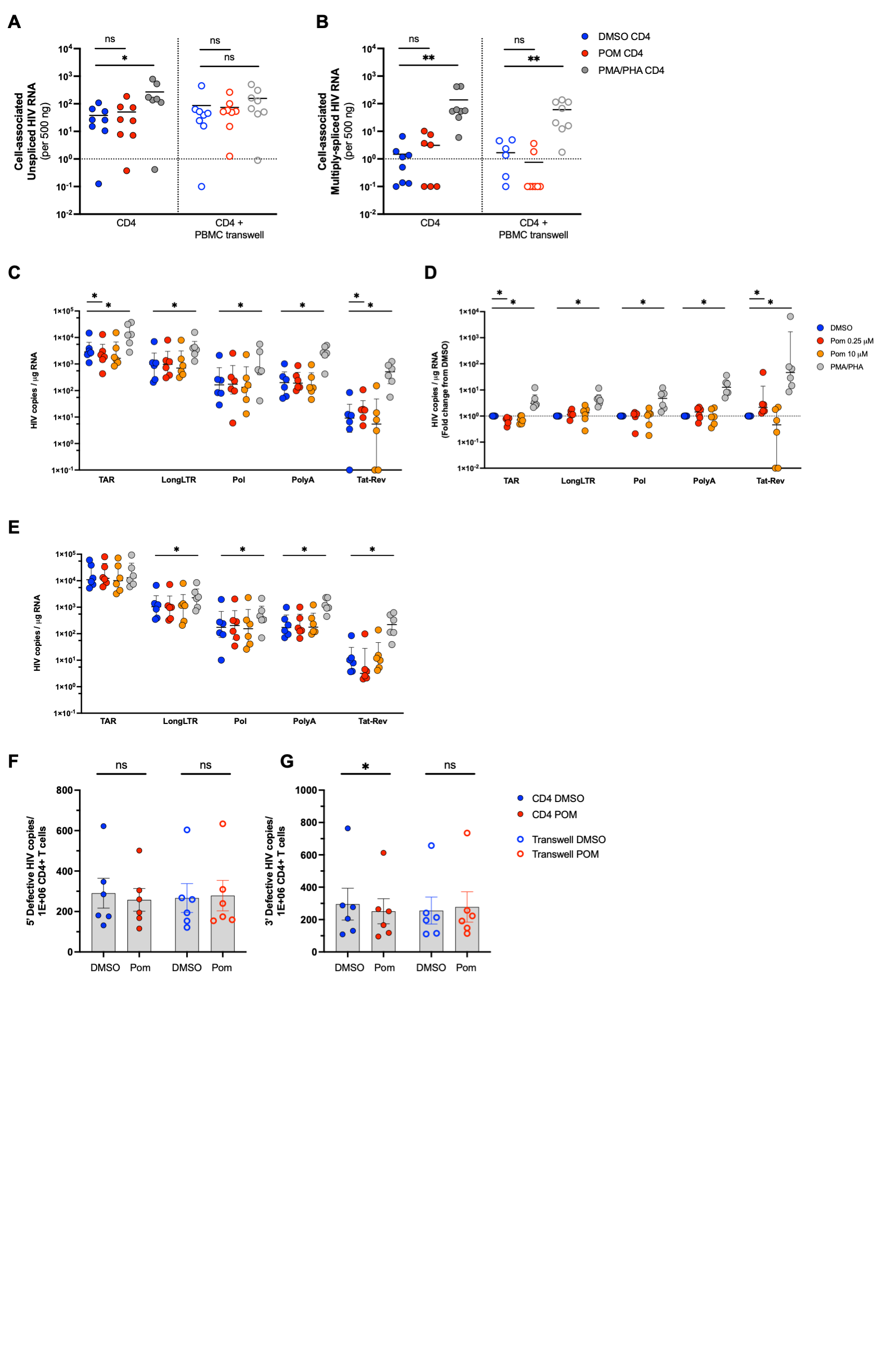

Supplement: Figure S5 — Pomalidomide does not induce latency reversal in CD4+ T cells from ART-suppressed PLHIV. [file jvi.01676-24-s0005.tiff]
