# Supplementary material for: Targeting Ikaros and Aiolos with pomalidomide fails to reactivate or induce apoptosis of the latent HIV reservoir
Source: J Virol. 2025 Feb 4;99(3):e01676-24. doi: 10.1128/jvi.01676-24 (PMC11915836; doi:10.1128/jvi.01676-24)
Supplement: Supplemental legends and tables — Legends for Fig. S1 to S5; Tables S1 and S2. [file jvi.01676-24-s0006.docx]

**Supplementary Figure 1. Pomalidomide degrades Ikaros and Aiolos in CD4+ T cells from PLHIV on suppressive ART after 24 and 72 hours treatment *ex vivo*.**

CD4+ T cells from PLHIV on suppressive ART were treated with pomalidomide (0.25 μM, 2 μM, 10 μM) or DMSO for 24 and 72 hours, and expression of Ikaros (IKZF1) and Aiolos (IKZF3) were measured in lysates after being subjected to polyacrylamide gel electrophoresis and measured using immunoblotting with antibodies against IKZF1 and IKZF3. Protein input was normalized between drug conditions, as evidenced by the GAPDH bands.

**Supplementary Figure 2. Pomalidomide upregulates stress protein expression on HIV-infected and HIV-uninfected CD4+ T cells.**

CD4+ T cells were infected *in vitro* with a green fluorescent protein (GFP) expressing HIV and then treated with pomalidomide at 0.25 μM or DMSO for 5 days. **(A)** Representative gating strategy shown, gating on **CD3^dim+high^** and **CD4^dim+high^ to capture HIV productively-infected CD4+ T cells. (B)** Representative histogram overlay of HLA-A/B/C, HLA-E, MIC-A/B, and ULBP-2/5/6 expression in *in vitro* HIV-infected (GFP+) and HIV-uninfected (GFP-) CD4+ T cells following treatment with pomalidomide at 0.25 μM, or DMSO, for 5 days; and then analysed for **(C)** average fold change in expression of HLA-A/B/C, HLA-E, MIC-A/B, ULBP-2/5/6, and CD155 on HIV productively-infected (GFP+) and HIV-uninfected/non-productively-infected (GFP-) CD4+ T cells following pomalidomide treatment relative to DMSO control. Average of 6 donors shown; **(D)** Representative flow plots of DMSO and pomalidomide-treated HIV-infected CD4+ T cells following co-culture with the NK cell line, KHYG1, for 4 hours, in the absence or presence of an anti-NKG2D blocking antibody (Ab); **(E)** Percentage killing of DMSO and pomalidomide-treated HIV-infected CD4+ T cells following co-culture at the effector:target (KHYG1: CD4+ T cells) ratio of 1:1. *p < 0.05; ns, not significant. Wilcoxon matched-pairs signed rank test.

**Supplementary Figure 3: Pomalidomide elevates CD155 and PD-L1, and reduces TIGIT expression on memory CD4+ T cells from ART-suppressed PLHIV.**

CD4+ T cells from PLHIV on suppressive ART were treated with pomalidomide at 0.25 μM, or DMSO for 72 hours *ex vivo;* **(A-B)** representative flow gating of **(A)** CD4+ T cells memory subsets and **(B)** surface marker expression of PD-1, PD-L1, TIGIT, TIM-3, CD57, and CD155, shown as a staggered overlay contour plot; **(C)** frequency of PD-1, PD-L1, TIGIT, TIM-3, CD57, and CD155 on CD4+ T cell memory subsets following treatment with DMSO (blue) or pomalidomide (red). *p < 0.05; **p < 0.01. Each symbol in Supp. Fig. 3C represents a single donor. Wilcoxon matched-pairs signed rank test. NA= naïve, CM = central memory, TM = transitional memory, EM = effector memory, TD = terminally differentiated CD4+ T cells.

**Supplementary Figure 4: In the presence of stimulation, pomalidomide enhances the proliferation of memory CD4+ T cells.**

CD4+ T cells from ART-suppressed PLHIV were treated with pomalidomide at 0.25 μM, or DMSO in the presence of T cell receptor (TCR) stimulation for 6 days, and analysed for **(A)** Frequency of proliferation in central memory (CM), transitional memory (TM), and effector memory (EM) CD4+ T cells following DMSO or pomalidomide treatment, with numbers showing the median fold change (MFC) of pomalidomide-treated conditions relative to DMSO; and **(B)** the proportion of T cell subsets (including naïve, NA, and terminally differentiated, TD) to the proliferating CD4+ T cell population (n=6). *p < 0.05; ns, not significant. Each symbol in Supp. Fig. 4A represents a single donor. Wilcoxon matched-pairs signed rank test.

**Supplementary Figure 5. Pomalidomide does not induce latency reversal in CD4+ T cells from ART-suppressed PLHIV.**

CD4+ T cells from ART-suppressed PLHIV were treated with pomalidomide at 0.25 μM, or DMSO for 72 hours *ex vivo*, and analysed for **(A)** cell-associated unspliced (US) HIV RNA per 500 ng and **(B)** cell-associated multiply-spliced (MS) HIV RNA per 500 ng; **(C-D)** CD4+ T cells from ART-suppressed PLHIV were treated with pomalidomide at 0.25 μM, 10 μM, phorbol 12-myristate 13-acetate (PMA)/phytohaemagglutinin (PHA) or DMSO for 24 hours *ex vivo*, and the HIV transcripts per 500 ng input were quantified by RT-PCR, including transcriptional initiation (TAR), elongation beyond the 5’ LTR sequence (Long LTR), polymerase (pol), completion of transcription (PolyA), and multiply-spliced Tat-Rev (Tat-Rev) HIV RNA, with **(C)** HIV copies/μg RNA shown and **(D)** HIV transcripts shown as a fold change of drug-treated relative to DMSO vehicle control; **(E)** HIV transcripts were measured in CD4+ T cells treated with pomalidomide or DMSO for 72 hours, with HIV copies/μg RNA shown; **(F-G)** CD4+ T cells from PLHIV on ART were treated with DMSO or pomalidomide for 72 hours. CD4+ T cells were cultured alone (solid circles) or in a transwell membrane separated from autologous PBMC for paracrine stimulation (open circles), and DNA extracted for quantification by PCR of **(F)** 5’ defective HIV DNA and **(G)** Hypermutated/3’ deletion (3’ defective) HIV DNA. *p < 0.05; **p < 0.01; ns, not significant: Wilcoxon matched-pairs signed rank test. Each dot represents a single donor. Bars show median + IQR.

Schematic created with BioRender.com.

**Supplementary Table 1. Clinical characteristics of study participants**

| Participant ID | Age (years) | Race | Years since HIV diagnosis | CD4+ count (cells/μL) | CD4  (%) | CD8+ count (cells/μL) | CD8 (%) | Nadir CD4+ count (cells/μL) | ART regimen | Duration HIV RNA<50 copies (years) |
| --- | --- | --- | --- | --- | --- | --- | --- | --- | --- | --- |
| PRA002 | 48 | Caucasian | 10 | 1460 | 47 | 793 | 26 | 698 | ABC/3TC, EFV | 12 |
| PRA003 | 49 | Caucasian | 19 | 590 | 31 | 767 | 29 | 218 | TDF/FTC, DRV, RTV | 15 |
| PRA004 | 55 | Caucasian | 20 | 1036 | 40 | 1069 | 42 | 266 | TAF/FTC, DTG | 15 |
| PRA009 | 49 | Caucasian | 9 | 474 | 25 | 1085 | 56 | 42 | EVG/TAF/FTC/COBI | 13 |
| PRA011 | 53 | Caucasian | 14 | 735 | 37 | 810 | 41 | 300 | ABC/3TC, EFV | 11.2 |
| ICB2161 | 69 | Caucasian | 33 | 800 | 42 | 647 | 34 | 98 | 3TC, DRV, RTV, DTG | 7 |
| ICB2208 | 66 | Caucasian | 34 | 466 | 31 | 546 | 36 | 54 | FTC/TAF, DRV/COBI | 9 |
| ICB2467 | 46 | Hispanic/Latino | 11 | 429 | 43 | 316 | 32 | 324 | RPV/TAF/FTC | 10.4 |
| ICB2651 | 52 | Caucasian | 17 | 655 | 37 | 681 | 39 | 275 | ABC/DTG/3TC | 14.0 |
| ICB3147 | 61 | Hispanic/Latino | 25 | 837 | 44 | 522 | 27 | 4 | ABC/DTG/3TC | 11.0 |
| ICB3162 | 56 | Caucasian | 31 | 586 | 37 | 471 | 30 | 200 | DRV,RTV, ABC/DTG/3TC | 11.5 |
| LKA04 | 56 | Caucasian | 29 | 767 | 35 | 530 | 24 | 230 | ABC/3TC/DTG | 3 |
| LKA12 | 57 | Caucasian | 20 | 744 | 37 | 932 | 46 | 624 | RAL, DRV | 3 |
| LKA15 | 42 | Caucasian | 11 | 681 | 39 | 698 | 40 | 198 | NVP, TDF/FTC | 10 |
| LKA16 | 67 | Caucasian | 11 | 534 | 39 | 315 | 35 | 315 | ABC/3TC/DTG | 10 |
| LKA18 | 60 | Caucasian | 25 | 312 | 19 | 312 | 34 | 2 | TAF/FTC | NA |
| LKA19 | 28 | Caucasian | 4 | 1072 | 42 | 857 | 33 | 731.9 | TAF/FTC/RPV | NA |
| n-Cure 120  13/02-20 | 59 | Caucasian | NA | 670 | 51.2 | NA | NA | NA | FTC, TAF, EVG, COBI | > 8 |
| Median  (IQR) | 55.5 (11.5) | NA | 19.0  (16.0) | 675.5  (290.3) | 38.0  (8.3) | 681.0  (337.0) | 34.0  (11.0) | 230  (243.5) | NA | 10.7  (4.5) |

*ART: Antiretroviral therapy; VL: viral load; NA: not available. ABC, abacavir; COBI, cobicistat; DRV, darunavir; DTG, dolutegravir; EFV, efavirenz; EVG, elvitegravir; FTC, emtricitabine; RPV, rilpivirine; RTV, ritonavir; TAF, tenofovir alafenamide; 3TC, lamivudine; TDF, tenofovir disoproxil fumarate*.

| Probes |  |  |  |  |
| --- | --- | --- | --- | --- |
| Name | 5’ Modification | Sequence | 3’ Modification | Supplier |
| Psi Probe | FAM | TTTTGGCGTACTCACCAGT | MGB | ThermoFisher |
| Env Intact Probe | VIC | CCTTGGGTTCTTGGGA | MGB | ThermoFisher |
| RPP30 Probe | VIC | CTGACCTGAAGGCTCT | MGB | ThermoFisher |
| Env Hypermutant Probe | - | CCTTAGGTTCTTAGGAGC | MGB | Integrated DNA Technologies |
| RPP30 Shear Probe | FAM | AAGGAGCAAGGTTCTATTGTAG | ZEN/3IABkFQ^c^ | Integrated DNA Technologies |
| Env2 Probe | VIC | CTGGCCTGTACCGTCAG | MGB | ThermoFisher |
| Primers |  |  |  |  |
| Psi Forward (Siliciano) |  | CAGGACTCGGCTTGCTGAAG |  | Sigma Aldrich |
| Psi Reverse (Siliciano) |  | GCACCCATCTCTCTCCTTCTAGC |  | Sigma Aldrich |
| Env Forward (Siliciano) |  | AGTGGTGCAGAGAGAAAAAAGAGC |  | Sigma Aldrich |
| Env Reverse (Siliciano) |  | GTCTGGCCTGTACCGTCAGC |  | Sigma Aldrich |
| RPP30 Forward |  | GATTTGGACCTGCGAGCG |  | Sigma Aldrich |
| RPP30 Reverse |  | GCGGCTGTCTCCACAAGT |  | Sigma Aldrich |
| RPP30-shear Forward |  | CCATTTGCTGCTCCTTGGG |  | Sigma Aldrich |
| RPP30-shear Reverse |  | CATGCAAAGGAGGAAGCCG |  | Sigma Aldrich |
| Env Forward (Jones) |  | ACTATGGGCGCAGCGTC |  | Sigma Aldrich |
| Env Reverse (Jones) |  | CCCCAGACTGTGAGTTGCA |  | Sigma Aldrich |

**Supplemental Table 2. IPDA primer and prob**
